# Single-Prolonged Stress: A Review of Two Decades of Progress in a Rodent Model of Post-traumatic Stress Disorder

**DOI:** 10.3389/fpsyt.2018.00196

**Published:** 2018-05-15

**Authors:** Michael J. Lisieski, Andrew L. Eagle, Alana C. Conti, Israel Liberzon, Shane A. Perrine

**Affiliations:** ^1^Department of Psychiatry and Behavioral Neurosciences, Wayne State University School of Medicine, Detroit, MI, United States; ^2^Department of Physiology, Michigan State University, East Lansing, MI, United States; ^3^Research and Development Service, John D. Dingell Veterans Affairs Medical Center, Detroit, MI, United States; ^4^Department of Neurosurgery, Wayne State University School of Medicine, Detroit, MI, United States; ^5^Department of Psychiatry, University of Michigan, Ann Arbor, MI, United States; ^6^Mental Health Service, Veterans Affairs Ann Arbor Healthcare System, Ann Arbor, MI, United States

**Keywords:** PTSD, single prolonged stress, anxiety, amygdala, hippocampus, prefrontal cortex, HPA axis, pre-clinical models

## Abstract

Post-traumatic stress disorder (PTSD) is a common, costly, and often debilitating psychiatric condition. However, the biological mechanisms underlying this disease are still largely unknown or poorly understood. Considerable evidence indicates that PTSD results from dysfunction in highly-conserved brain systems involved in stress, anxiety, fear, and reward. Pre-clinical models of traumatic stress exposure are critical in defining the neurobiological mechanisms of PTSD, which will ultimately aid in the development of new treatments for PTSD. Single prolonged stress (SPS) is a pre-clinical model that displays behavioral, molecular, and physiological alterations that recapitulate many of the same alterations observed in PTSD, illustrating its validity and giving it utility as a model for investigating post-traumatic adaptations and pre-trauma risk and protective factors. In this manuscript, we review the present state of research using the SPS model, with the goals of (1) describing the utility of the SPS model as a tool for investigating post-trauma adaptations, (2) relating findings using the SPS model to findings in patients with PTSD, and (3) indicating research gaps and strategies to address them in order to improve our understanding of the pathophysiology of PTSD.

## Introduction

Post-traumatic stress disorder (PTSD) is a psychiatric disorder that develops following direct or indirect exposure to an extremely stressful (traumatic) event or series of events. Symptoms of PTSD include intrusive memories related to the traumatic event, distress in response to trauma-related cues and avoidance of those cues, negative alterations in cognition and mood, and increased arousal and reactivity (1). PTSD is diagnosed when these symptoms last longer than 1 month and cause functional impairment and distress. It's estimated that 3.5% of the US population (more than 11 million Americans) suffer from PTSD in a given year, but less than half of these patients are in treatment, and less than half of those in treatment receive minimally adequate care (2) receiving treatment may not experience remission; the two pharmacotherapeutics currently approved by the US Food and Drug Administration for treatment of PTSD have relatively limited efficacy (3) and focus on reducing symptoms rather aimed at the underlying pathophysiology of PTSD. Given the high prevalence and significant health burden of PTSD, research aimed at better understanding its biological basis and using this knowledge to develop efficacious treatments is absolutely essential.

Because our ability to examine the neurobiology of PTSD in humans is limited, pre-clinical models provide a valuable opportunity to investigate the effects of traumatic stress exposure. The goal of this review is to provide an overview of research conducted using one pre-clinical model of PTSD, the single prolonged stress (SPS) model, and to examine its validity, utility, and potential for contributing to new and improved therapies for PTSD. While we focus on the SPS model, several other pre-clinical models have been proposed as relevant for PTSD. These include Pavlovian fear conditioning (4, 5), stress-enhanced fear learning (6, 7), exposure to predators or predator scent (8–10), physiological stressors such as underwater trauma (11), or restraint stress (12, 13), and protocols that (like SPS) combine multiple stressors, for example the combination of social instability with predator exposure (14) or social isolation with foot shock exposure (15). Several recent reviews have evaluated and compared these models (16–22); however, making comparisons among them is not the primary aim of this review. Rather, after briefly describing the symptomatology of PTSD and key brain systems involved in this disorder, we review studies that demonstrate the utility of the SPS model to expand our understanding of clinical PTSD and the molecular mechanisms that may underlie it, and discuss potential avenues for research and novel treatment targets. In addition, a condensed summary of findings regarding the behavioral and neurobiological effects of SPS is given in Supplementary Tables 1, 2.

## Symptomatology of PTSD

The American Psychiatric Association's Diagnostic and Statistical Manual of Mental Disorders (DSM 5), identifies four clusters of PTSD symptoms: intrusive re-experiencing of the traumatic event(s), avoidance of trauma-related thoughts and reminders, negative alterations in cognition and mood, and alterations in arousal (1). This clustering of PTSD symptomatology has empirical support; studies analyzing the factor structure of PTSD symptoms among diverse clinical populations such as victims of intimate partner violence (23), refugees (24), and earthquake survivors (25) provide evidence for factor structures in PTSD symptomatology which are broadly consistent with (if not identical to) the DSM 5 model.

Translating clinical diagnostic criteria and patient characteristics to constructs that can be operationalized and used in pre-clinical behavioral research is a perennial challenge. The Research Domain Criteria developed by the National Institute of Mental Health (26) attempts to provide parsimonious definitions of neurobehavioral phenotypes that are relevant to psychiatric disorders, each of which can be linked to specific behaviors, cellular, and molecular processes. We take a similar approach, grouping findings in SPS research by behavioral domain.

Only subsets of PTSD symptoms readily map onto behavioral domains that can be modeled in non-humans. For example, assessing nightmares, self-blame, and the presence of trauma-related thoughts rely on self-report, and therefore, cannot be modeled in non-humans. However, the majority of behavioral domains relevant to PTSD symptoms can be studied in non-humans in some form; these include exaggerated startle response, disruption of sleep and circadian cycle, and avoidance or fear of trauma-related cues. Increased generalization and persistence of conditioned fear, while not part of the DSM5 diagnostic criteria, has been repeatedly observed in humans with PTSD (27–29), presumably involves mechanisms underlying the persistence of traumatic memories, and is readily studied in non-humans (30). Also experimentally accessible are characteristics of PTSD which are usually self-reported in humans but can also inferred through behavioral testing, such as changes in anxiety-like behavior and threat assessment, constriction of affect and reward, and disruption of attention and cognitive performance. Therefore, pre-clinical neurobehavioral analyses provide powerful tools for studying most, though not all, key behavioral processes disrupted in PTSD.

## Neuroanatomy of PTSD

Neuroimaging studies have identified a set of brain regions that likely contribute to the behavioral abnormalities in PTSD, including the hippocampus (HC), amygdala (AMY), and prefrontal cortex (PFC) (31–33). This triad of brain regions is also central to the brain circuit implicated in fear/safety learning (34). The function of the hypothalamic-pituitary-adrenal (HPA) axis also plays a key role in stress response (35) and has repeatedly been demonstrated to be altered in PTSD (36). As many clinical and pre-clinical studies on PTSD investigate these systems in some way, we will briefly describe their structure and function and review evidence of their involvement in PTSD from clinical and human laboratory studies.

### Hippocampus

The HC is a subcortical structure that plays a critical role in learning and memory (37), and integrates contextual information to regulate behavior (38). The HC therefore stands out as a likely substrate for memory-related problems in PTSD, which include persistent re-experiencing of traumatic events, increased salience of negative emotional memories (39), deficits in working and verbal memory (40–42), and impaired context-dependent modulation of memory (31, 43). Subregions of the HC are functionally heterogenious; studies in rat show that the dorsal HC (homologous to the human posterior HC) is primarily involved in information processing and cognitive performance whereas the ventral HC (homologous to the human anterior HC) is important in regulation of stress response and affect (44).

Hippocampal volume is reduced in individuals with PTSD compared to controls; however, reduced HC size is also seen in individuals exposed to severe stressors with or without PTSD (45, 46) and in non-trauma exposed twins of individuals with PTSD (47). Additionally, larger HC size is related to a positive response to PTSD treatment (48). These findings suggest that the link between HC size and PTSD diagnosis results both from vulnerability conferred by having a small HC and from a reduction in HC size caused by traumatic stress exposure. Corroborating structural studies, functional neuroimaging studies have shown altered HC function in individuals with PTSD (49). The HC has reciprocal connections to many other brain areas; importantly, the HC interacts with the PFC to regulate memory (50, 51) and with the AMY to regulate emotional arousal (52) and consolidation of fear memories (53). HC connectivity is uniquely altered in PTSD (54) and HC connectivity can predict symptom severity in patients with PTSD (55), suggesting that these connections may underlie PTSD-related deficits and emphasizing the importance of the HC in PTSD pathophysiology (54, 55).

### Prefrontal cortex

The PFC encompasses a large area in the frontal lobe of the brain and has roles in decision-making, “executive” functions such as attention and working memory, and regulation of emotion (56, 57). Importantly for PTSD, the PFC plays a role in the regulation of fear learning, expression, and extinction (58). The function of two PFC subregions, the anterior cingulate cortex (ACC) and the medial PFC (mPFC), has been found to be altered in PTSD (59, 60). These brain regions regulate affective responses generated by other brain regions. Of great importance for understanding PTSD and its treatment, the human ventromedial PFC (vmPFC; analogous to the infralimbic cortex, IL, in rodents) plays a critical role in the extinction of fearful memories by processing safety signals (61) and interacting with the AMY to inhibit fear expression (62). Correspondingly, disruption of vmPFC function appears to contribute to altered emotional processing (63) and impaired retention of fear extinction learning (28, 29, 64) in PTSD.

### Amygdala

The AMY is often considered “a hub” of emotional processing. Of particular relevance to PTSD, the AMY plays an important role in fear learning and extinction (65). Sensory information primarily flows into the basolateral AMY (BLA), where long-term potentiation key to associative fear learning takes place, and the signal is then conveyed to the central AMY (CeA) which regulates the output of fear behavior (65). The AMY receives input from brain regions such as the IL and sensory areas, which may act as “gates” that inhibit expression of learned fears (66). Studies in clinical populations with PTSD have found that AMY response to emotional stimuli is exaggerated in individuals with PTSD (67) and that AMY responses to fearful stimuli predict treatment response (68). These results and associated pre-clinical results indicate that dysfunction of the AMY and its connections with other brain regions may underlie the excessive persistence of fearful memories and other emotional symptoms of PTSD.

### Other brain regions

Brain regions outside of the HC-AMY-PFC circuit described above have also been implicated in PTSD. Insular cortex function, for example, is altered in PTSD and anxiety-related disorders (69) and has roles in processes relevant to PTSD such as affective processing and interoceptive awareness (70). Despite this role, no studies using the SPS model have yet directly investigated insula structure or function.

Evidence that individuals with PTSD have increased startle response (71) and reduced magnitude of P3 (72), an event-related potential that may reflect increased noradrenergic output (73), suggests that locus coeruleus (LC) function might be altered in PTSD. Building on this work, several studies have investigated the effects of SPS on LC function.

Finally, evidence for dysfunction of reward and hedonic processes in PTSD (74) converges with neuroimaging evidence that striatum dysfunction is related to the affective and cognitive symptoms of PTSD (75, 76). Changes in striatal function may also explain the links between PTSD and substance use disorders (SUD) (77), compulsive behaviors (78–80), and risk-taking (81, 82). Several studies have investigated striatal function following SPS, largely in relation to its role in responses to drugs of abuse and other extrinsic rewards.

### HPA axis

A great deal of research has focused on the HPA axis in PTSD. The HPA axis coordinates stress responses through a highly regulated neurohormonal cascade. Corticotrophin releasing hormone (CRH) is released from hypothalamic neurons, liberating adrenocorticotropic hormone (ACTH) from pituitary cells, which in turn stimulates the secretion of cortisol (CORT) into the bloodstream by the adrenal cortex. CORT, in turn, has effects on a wide variety of tissues, including activating glucocorticoid receptors (GR) and mineralocorticoid receptors (MR) in the HC, AMY, hypothalamus, pituitary, and other regions of the brain. Activation of hypothalamic GR receptors decreases HPA axis activity, creating a negative feedback loop that limits the persistence of stress responses. The HPA axis also interacts with the brain non-hormonally; the paraventricular nucleus of the hypothalamus, where CRH-secreting cells are located, has connections with, and is regulated by, other brain regions implicated in PTSD, such as the HC (83) and AMY (84, 85). These brain regions participate in both feedback inhibition and feed-forward stimulation of the HPA axis to regulate stress responses (86). Particularly severe or persistent stressors can cause long-lasting changes in the function of HPA axis and brain responses to CORT and CRH, and this “stress programming” may contribute to the variety of maladaptive effects that severe stress can have on individuals (85, 87, 88).

## Single prolonged stress (SPS)

SPS is a multimodal traumatic stress exposure protocol including sequential exposure to three stressors (2 h of restraint, a 20-min group swim, and exposure to ether until loss of consciousness) during a single continuous session. This protocol was originally designed to cause a robust stress response through three different pathways—psychological (restraint), physiological (forced group swim), and pharmacological (ether). SPS was originally described by Liberzon and Young (89) as “time-dependent [stress] sensitization,” and was observed to cause an abnormal phenotype characterized by enhanced fast negative feedback of the HPA axis which was emerging 7 days, but not 1 day, after stress exposure. This incubation period, which has also been called a sensitization or consolidation period, is usually 7 days in length. While some studies have investigated post-SPS neurobiological and behavioral changes before and after this time point, more studies are needed to define the temporal development and persistence of PTSD-like characteristics following SPS. Later work revealed similar behavioral and neuroendocrine effects in mice exposed to a similar procedure (90), demonstrating the viability of this approach in multiple rodent species. The specificity of the phenotype and the time-dependent nature of the change corresponds to the altered neuroendocrine response to stress in individuals with PTSD (91–93) and the dynamic course of PTSD symptoms, which tend to escalate over time to produce long-term functional impairment (94, 95). Further studies demonstrated that combined exposure to all three components of SPS is required for the expression of PTSD-like phenotypes (96), suggesting that interaction among the multiple components of SPS induces abnormal neurobiological phenotypes associated with PTSD differently than any component stressor alone (23–25, 97). Over the past 20 years, SPS had been widely used to study adaptations following a severe traumatic stressor, with great emphasis placed on characterizing behavioral changes following SPS and their relationship to underlying neurobiological processes.

In order to provide a useful platform for studying post-trauma adaptations relevant to PTSD, the SPS model (and any pre-clinical model) must satisfy few basic criteria. In particular, a model of PTSD should recapitulate the phenomenology of the disorder by producing behavioral changes that resemble behavioral changes seen in PTSD, and which rely on similar mechanisms (18). Furthermore, interventions that improve PTSD symptoms should also improve corresponding behavioral changes in the model, and vice versa. SPS has been demonstrated to satisfy these conditions of validity (97), as it uses a single episode of severe stress to generate persistent behavioral changes in PTSD-relevant behavioral domains which are responsive to interventions used to treat PTSD.

The remainder of this review describes recent progress that has been made in using SPS to understand the pathophysiology of PTSD. Where appropriate, we include brief comparisons to research on human patients to emphasize the validity of SPS as a model of traumatic stress.

## Behavioral effects of SPS

### Abnormal fear learning

PTSD has long been proposed to involve aberrant function of normal fear learning processes (98) leading to abnormal responses to potential threats and non-threatening (but trauma-associated) stimuli (99). This has led researchers using SPS to focus on behaviors and brain mechanisms involved in fear memory formation and retrieval (100). Several phases of fear conditioning can be studied: the acquisition of a fear memory, the expression of conditioned fear after a delay, the extinction of the conditioned fear response, the retention (recall) of this extinction, and the reactivation of an extinguished fear response through spontaneous recovery, reinstatement, or renewal (101). While fear conditioning alone is not a model of PTSD, it is used to assess abnormalities in fear learning that are associated with PTSD. In experimental studies that use PTSD models, fear conditioning can be broadly categorized into two paradigms: trauma cue-specific fear conditioning wherein a neutral stimulus that was paired with the trauma exposure is used to reactivate memory of the traumatic event, and *de novo* fear conditioning in which a neutral stimulus (cue or context) is paired with a novel aversive stimulus, with this whole process taking place after exposure to a traumatic event.

#### Trauma-cue responses

One of the defining characteristics of PTSD is increased reactivity to and avoidance of cues associated with the traumatic stressor (1). This has been investigated in a number of human laboratory studies (102), it was even proposed as potential biomarker to predict (103) and index (104) treatment response. There are relatively few reports on the effects of SPS on trauma cue-specific fear despite the fact that SPS, as a procedure consisting of a single exposure to a strongly aversive event, is amenable to this type of experimental design.

Defensive reactions to and avoidance of trauma cues have been demonstrated for up to 43 days following SPS exposure in rats (105) and up to 7 days following SPS exposure in mice (90). This has been observed to occur to both direct trauma-associated cues (e.g., restraint apparatus, swim tank, ether chamber) and peripherally-associated cues (e.g., holding chambers, tones, scents) (90, 105). Additionally, while the fear response appears to extinguish following repeated exposure to a trauma-associated scent cue, it can be reinstated by exposure to the anxiogenic drug yohimbine (106), suggesting that the extinguished response remains sensitive to reactivation. Further studies are needed to determine the neuronal underpinnings of trauma cue reactivity in SPS and how this differs from standard fear conditioning protocols.

#### De novo fear conditioning

When individuals with PTSD undergo extinction learning, this extinction is not as well-retained as in control subjects; thus, it is said that these individuals with PTSD have impaired extinction retention (28, 29, 107). This could be due to trauma-induced neurobiological changes in PTSD (28), or may reflect a pre-existing impairment in memory that predisposes susceptible individuals to PTSD following trauma exposure (108). Mirroring clinical findings, SPS produces robust extinction retention deficits in rats trained to associate cues (auditory tones) with foot shock (96, 109–114), and in mice exposed to SPS (90). A similar pattern of delayed or poorly-retained extinction is also demonstrated by some studies of contextual fear conditioning following SPS, in which an environmental context serves to predict the shock rather than a discrete cue (115–,117). The effect of SPS on extinction retention is time-dependent, requiring an incubation period to emerge (111). SPS-induced extinction retention deficits have been associated with enhanced GR expression in the HC (96) and decreased activity in the IL during fear recall (118).

SPS also enhances the magnitude of conditioned fear response to contexts associated with aversive stimuli such as foot shock in rats (114, 119–128) and mice (129). This enhanced contextual fear conditioning has been observed to recover by 4 weeks following SPS (130), in line with the clinical finding that populations exposed to some kinds of traumatic events (non-intentional traumatic events, in particular) tend to express symptoms initially but gradually recover spontaneously without treatment (131). Conditioned taste aversion, while not a type fear learning, is also enhanced following SPS (132), indicating that memory for aversive events may be abnormally high following trauma across modalities and tasks.

Finally, PTSD may produce specific dysregulation of contextual processing of aversive stimuli, leading to overgeneralization of learned fear and impaired ability to learn or respond to safety signals. Clinical research using fear conditioning paradigms demonstrates that PTSD patients do not adequately use context or cues to distinguish between threatening signals (those which indicate that an aversive stimulus is likely to occur) and safety signals (those which indicate that an aversive stimulus is unlikely to appear) (31, 133). This has been associated with the tendency to over-generalize negative contextual associations (43). Furthermore, clinical studies suggest that overgeneralization of conditioned fear may predict treatment responsiveness (27), indicating the importance of understanding the effects of traumatic stress on fear generalization.

In contrast to studies in humans showing a reduction of discrimination between fearful and non-fearful contexts in humans with PTSD (31), contextual fear discrimination was intact in SPS-exposed rats in some studies (122). However, we have recently shown that a subset of susceptible mice exposed to SPS have impaired fear discrimination in a cue-conditioning task, indicating that they generalize conditioned fear to non-threatening stimuli more readily than control animals (30).

Studies using *de novo* fear conditioning provide ample evidence that SPS mirrors fear deficits observed in PTSD, providing support for the model's applicability and translational relevance. Further research is necessary to determine what factors predispose trauma-exposed individuals to maladaptive disruptions of fear learning both in regard to trauma-cue reactivity and abnormalities in *de novo* fear conditioning. SPS, as a well-validated pre-clinical model of post-traumatic alterations in fear learning, can serve as a powerful platform to study the mechanisms underlying these changes, which can inform translational work aimed at improving treatments for PTSD.

### Hyperarousal

Hyperarousal is one of the criteria of PTSD as defined by the DSM 5 (1). Symptoms that indicate hyperarousal include exaggerated startle response, hypervigilance, difficulty concentrating, and disruption of sleep. Several behaviors related to these symptoms are amenable to testing in non-human models. Exaggerated startle response, in particular, has been studied relatively extensively following SPS. Indices of cognitive performance that rely on attention and memory have also been studied following SPS, as have neurophysiological aspects of sleep; additionally, anxiety-like defensive behavior observed in rodents may correspond to the hypervigilance reported by patients with PTSD, as factor analyses of PTSD symptoms have identified an “anxious hyperarousal”-like factor that includes endorsement of both anxiety and arousal items (23–25). Effects of SPS on cognitive performance, sleep, and anxiety-like behavior are discussed in subsequent sections.

The startle response is used to assess anxiety and hypervigilance across many species and reflects sensitivity to potential threat (134). PTSD commonly enhances startle responses (71, 135, 136), indicating that this may be a useful translational tool to investigate the hyperarousal that often characterizes PTSD (137). SPS has been repeatedly confirmed to increase startle response (105–140), and specifically only after an incubation period (122). Exaggerated acoustic startle has been used as tool for the evaluation of PTSD treatments in the SPS model; for example, the anti-epileptic drug, topiramate, which may be useful in treating PTSD (141), was shown to attenuate SPS effects on startle response (140). Other classes of drugs including cannabinoid agonists (138), neuropeptide Y (NPY) (139), and psychostimulants (105) also blocked SPS effects on startle, indicating that drugs that target these neurotransmitter systems may be efficacious treatments for post-traumatic hyperarousal.

### Cognitive dysfunction

PTSD is often associated with more general cognitive dysfunction, including memory impairment (40), deficits in mental processing speed (142), and inattention (143). Cognitive impairment may be a result of PTSD pathophysiology, and/or it may be a risk factor for PTSD, as clinical evidence suggests that deficits in executive function may be detected in individuals who will go on to develop PTSD following trauma exposure (144–146). Similarly, SPS induces impairments in attention and non-fear memory performance. For example, SPS decreases learning performance in a spatial water maze (122, 147). However, the interpretation of these results is confounded by re-exposure to water, which is a reminder of the stressor in SPS-exposed rats but not controls (106) and so may affect performance in this task. We and others have also observed impairment in object recognition (148, 149) and social discrimination (148). This impairment in social discrimination may be attributed to decreased behavioral flexibility, a notion corroborated by evidence that SPS decreases performance in set shifting tasks (150–152). These studies are in line with evidence that SPS reduced spontaneous activity in the LC (153) and altered mPFC function (118, 154), as both the LC (155) and mPFC (156) are important in maintaining attention and cognitive flexibility.

### Sleep

Sleep disruption likely contributes to the development and maintenance of PTSD in humans (157), and is associated with diverse aspects of PTSD pathology, including sustained fear responses to trauma cues (158), hyperarousal (159), increased use of cannabis (160), and suicidality (161). Therefore, understanding the role of sleep in PTSD is necessary to identify post-trauma adaptations leading to novel targets for PTSD treatment. Mirroring findings on disrupted sleep architecture in patients with PTSD (162), SPS causes increased wakefulness and REM sleep (163–165). Importantly, the degree of sleep disruption predicted SPS-induced extinction retention deficits (113), which suggests that traumatic stress-induced sleep alterations likely contribute to other PTSD symptoms.

### Anxiety

While general anxiety is not a defining characteristic of PTSD in the DSM5, increased anxiety is often reported by individuals with PTSD (166). Sustained defensive (unconditioned anxiety-like) behaviors are reliably measured in rodents using standard behavioral assays, including the elevated plus maze, open field, and light-dark box. A large number of reports show that SPS produces or increases anxiety-like behavior in these established behavioral models of anxiety (120, 124, 130, 167–173). Studies using modifications to SPS, such as re-exposure to stressors (147) or the addition of other stressors to the SPS paradigm (129, 139, 174–179), also report increased anxiety-like behavior. On the other hand, other studies fail to find a consistent anxiety-like phenotype produced by SPS (180–184). One powerful strategy to determine the relationship between anxiety-like behavior and more canonical PTSD-like phenotypes is to identify subpopulations of individuals that express anxiety-like behavior following traumatic stress and quantify other stress-related behaviors in these individuals to determine whether anxiety-like behavior changes in concert with other behaviors affected by SPS. Employing this approach, recent studies have identified that animals with high post-SPS anxiety also show exaggerated response to trauma cues (105). Furthermore, the time course of anxiety following SPS may be different from other phenotypes, as it has been found that anxiety-like behavior recovers toward baseline within 4 weeks following SPS (130).

Despite the wide use of anxiety-like behavior as a marker of “PTSD-like” changes following SPS, there are no published reports of the effects of SPS on some brain regions important in anxiety-like behavior (for example, the bed nucleus of the stria terminalis and insula), even though these brain regions have been previously implicated in the pathology of PTSD, for example in human neuroimaging studies (185) and in rodent studies on stress sensitization using other animal models (186, 187). Future research is necessary to relate specific post-SPS behavioral changes to neuronal systems outside of the HC-mPFC-AMY circuit and HPA axis involved in PTSD, including studies that differentiate populations which are uniquely affected by stress. This approach may be productive in identifying specific treatment targets for subgroups of PTSD patients, such as those who show treatment resistance to standard medications, those with high anxiety, and those with persistent (rather than spontaneously regressing) symptoms of PTSD (19).

### Disruptions of affect and reward

Negative changes in affect comprise part of the current diagnostic criteria for PTSD (1); this symptom cluster is sometimes referred to as “emotional numbing” and includes amnesia, anhedonia, and detachment (188). Reactivity to natural rewards and motivation to pursue them are both readily measured in pre-clinical rodent models, making these behavioral domains amenable to testing following SPS.

Negative affective disturbances including anhedonia and decreased motivation are commonly reported by PTSD patients (189). We have repeatedly observed decreased sucrose preference following SPS (169, 190). While these findings are suggestive of anhedonia, they do not distinguish between affective response and motivation, as changes in either of these processes could lead to reduced consumption of a palatable substance. Demonstration of reduced positive affective responses awaits future research using more specific analyses such as facial reactivity analysis (191) or measurements of reward-related ultrasonic vocalization (192).

Learned helplessness, a putatively affective disruption typically associated with depression (193), is increased following SPS in the water maze (194) and forced swim test (97, 139, 177, 178, 195). Importantly, increased learned helpless in the forced swim test models may model learned helplessness associated with depression (196) and is reversed by antidepressants (197). However, the use of forced-swim tests following SPS is likely confounded in that water acts as a potent trauma-associated cue (106) and stressor (132, 183, 198) in its own right. Supporting this, SPS did not affect a similar measure of learned helplessness that involves no water exposure, the tail suspension test (97). Additionally, forced swim test behavior changes over multiple exposures to water (199), and so post-SPS changes may simply reflect adaptive habituation rather than a maladaptive change (200, 201). Therefore, future studies into the effects of SPS on learned helplessness-like behavior should include test procedures that do not themselves serve as trauma cues, such as tail suspension (202), and exposure to inescapable shock (203, 204).

### Modeling comorbid conditions using SPS

While PTSD can be debilitating on its own, it is also highly comorbid with other psychiatric disorders. For example, SUD are disproportionately common among patients with PTSD, and this comorbidity is associated with increased mortality and poor treatment response (205, 206). Individuals with PTSD are also at increased risk for chronic pain (207, 208) and somatic disorders such as cardiovascular disease, respiratory disease, gastrointestinal disorders, and a variety of autoimmune disorders (208, 209). While a growing number of studies have investigated inflammation following SPS (see section Inflammation and Glia), none have yet directly modeled comorbid immune disorders. However, SPS has been used to investigate mechanisms related to substance use and pain perception following traumatic stress.

#### Substance use

Individuals with PTSD are prone to increased use of drugs including alcohol, nicotine, opioids, anxiolytics, marijuana, and psychostimulants (77, 205, 210–212). This may be due to a number of factors, including self-medication for PTSD symptoms, increased vulnerability to stress following substance use, or the presence of shared risk factors for PTSD and SUD (213). SPS, when combined with models of substance use, provides a powerful pre-clinical platform to investigate these processes and their neural underpinnings. Our initial studies utilized drug sensitization models to probe neurochemical changes following SPS, and more recent studies have combined self-administration models with SPS to gain insight into the biological relationships between SUD and PTSD.

Drug sensitization is a process by which the brain becomes more responsive to the presence of a drug over repeated use, which is a useful model for probing mechanisms regulating addiction (214). Drug sensitization is altered following SPS, although findings vary by the substance investigated. For example, we have shown that SPS blunts ethanol-induced behavioral sensitization (215), but enhances behavioral sensitization to repeated cocaine (216) and methamphetamine (217). Another group reported that SPS produced noradrenergic-dependent cross-sensitization to acute amphetamine (218). These interactions between SPS and drugs of abuse have been attributed to alterations in the norepinephrine system (218), dopaminergic (D2) receptors (190, 215), CB type 1 (CB1) receptors (215), and a marker of glutamatergic synapses (PSD-95) (215) in the striatum, indicating that they may result from diverse and widespread post-SPS changes in neurobiology.

In line with the hypothesis that traumatic stress exposure augments drug seeking and taking, SPS enhances ethanol reward (176). However, SPS, somewhat surprisingly, has no effect on short-access self-administration, extinction, and reinstatement of cocaine, and decreases cocaine conditioned place preference and long-access self-administration (190, 216). Experiments investigating other aspects of self-administration behavior, including motivation and drug sensitivity, need to be considered in future studies looking at drug seeking and taking behaviors in order to better understand the deficits caused by traumatic stress exposure on the reward system.

While findings that SPS alters drug sensitization and self-administration are broadly aligned with clinical findings of altered substance use in PTSD, it is as yet unclear how the mixed results could be synthesized. In resolving this issue, it will be imperative to consider individual differences in substance use responses after trauma exposure (219). An approach that identifies SPS-susceptible and resistant subpopulations has been used to show that a single injection of amphetamine can block expression of PTSD-like behaviors in PTSD-susceptible rats (218). Such heterogeneity is clinically meaningful; for example, PTSD patients with alcohol use disorder tend to have greater hyperarousal symptoms than those using other drugs (220), indicating that subpopulations of trauma-exposed individuals may have a higher propensity to self-administer specific drugs of abuse and may respond differentially to treatments. Further investigations of a wider variety of substances of abuse in SPS-exposed animals using sophisticated approaches to analyze heterogeneity among these animals provides an opportunity to study the converging effects of PTSD and SUD on brain function rigorously and robustly.

While several studies have investigated how SPS exposure affects responses to drugs of abuse, only one study has investigated the effects of exposure to an addictive drug on later responses to SPS, finding that cocaine did not enhance vulnerability to anxiety-like behavior after SPS (180). Since clinical findings indicate that many patients with both PTSD and SUD acquire SUD first (221), further studies investigating how a history of exposure to drugs of abuse may modulate the effects of SPS on other behaviors, and how pre-existing traits may influence the effects of both SPS and drug exposure, are important to thoroughly model the causal interactions between SUD exposure and the development of PTSD.

#### Enhanced pain sensitivity

Chronic pain syndromes are often comorbid with PTSD (222). However, clinical studies have fallen short of demonstrating the effects of PTSD on pain thresholds, emotional response to pain, and a potential shared vulnerability to chronic pain (223). This highlights the need for a pre-clinical model like SPS to investigate these phenotypes. Indeed, while there is some evidence to suggest that SPS decreases pain sensitivity (120), others have reported long-lasting *increases* in sensitivity using the same assay (224) and increased visceral hyperalgesia (225). Pre-clinical studies using the SPS model have also suggested mechanisms by which traumatic stress may enhance pain sensitivity, such as altered PKCγ in the spinal cord (225), enhanced reactivity of LC to noxious stimuli (153), and modulation via nociceptin/orphanin FQ signaling (224). These, and potentially other novel pathways, are promising targets for the treatment of chronic pain in patients with PTSD which continue to be investigated using SPS.

### Individual differences in vulnerability to SPS

As only a subset of individuals exposed to trauma develops PTSD, an important goal of research is to identify risk factors, including pre-existing or post-trauma factors (including age, sex, genetic, and personality differences, and early post-trauma response), that confer susceptibility or resilience to PTSD. To model pre-existing differences, such factors must be identified by manipulating or measuring relevant phenotypes before and after SPS exposure in longitudinal studies. Initial studies using such an approach have shown that greater pre-SPS anxiety predicts post-SPS enhanced reactivity to trauma-related cues (105, 106). In addition to considering pre-existing risk factors, individual variability in responsivity to trauma can be accounted for by differentiating SPS-exposed animals into susceptible and resilient populations using well-validated, replicable behavioral tests. For example, rats categorized as “vulnerable” to SPS using measures of enhanced anxiety and arousal also exhibit anhedonia, locomotor depression, and avoidance of trauma-related cues in a separate set of behavioral tests, whereas animals who are “resistant” to the effects of SPS on anxiety and arousal do not (106, 226). Future research using SPS should aim to take advantage of such longitudinal approaches to identify possible risk factors for PTSD, examine variability in responses to traumatic stress between “resilient” and “vulnerable” individuals, and identify the neural mechanisms that underlie these individual differences in traumatic stress responses.

In addition to identifying pre-existing or early-post-trauma traits that predict the enduring behavioral effects of traumatic stress, the effects of sex, age, and life history can be investigated using SPS. There is strong evidence that women are at increased risk for PTSD (206, 227) and experience symptoms differently than men, reporting greater distress, sleep disturbance, and diminished interest in daily activities (228–230), and exhibiting less maladaptive coping behavior (231). Evidence from human studies suggests that sex hormones such as estrogen may affect stress reactivity (232); however, many questions remain unanswered, particularly in the SPS model. The only report of sex differences following SPS found that SPS did not induce fear extinction retention deficits in female rats as it did in male rats, though it did alter GR expression in the dorsal HC of female rats (109). While two studies have shown that estradiol administration mitigates the behavioral effects of SPS, one study used only male rats (125) and the other did not report the sex of its subjects (126). These findings are therefore difficult to interpret, and further research on the role of sex and sex hormone in the SPS model are important in establishing its generalizability across sexes.

Epidemiological evidence indicates that age of trauma exposure interacts with other characteristics to determine likelihood of developing PTSD following a traumatic experience (233). Therefore, it is important to determine the role of age in the development of SPS. However, to date, no peer-reviewed publications have evaluated age as a factor influencing susceptibility to SPS. Clinical evidence also indicates that early life stress and environmental adversity contribute to the vulnerability to later life PTSD (234). Supporting this, one study found that neonatal isolation interacts with adult SPS by promoting greater SPS-induced increases in anxiety and contextual fear (120). The lack of published studies regarding the effects of age and sex on post-SPS behavioral and neurobiological changes highlights the need for research investigating age, sex, and previous life history on post-SPS outcomes, which could define neurobiological mechanisms which control susceptibility to PTSD.

## Neural and molecular mechanisms in SPS

### Neuroendocrine responses and corticosteroid receptors

HPA axis dysregulation is observed in many cases of clinical PTSD, making it an attractive potential biomarker for PTSD (137). While some studies indicate that basal CORT levels are low in PTSD (235, 236), differences in basal CORT levels between PTSD patients and controls are not consistently seen (237–239). A more reliable measure of HPA axis dysregulation is increased suppression of ACTH (91) and CORT (92) secretion in response to central stimulation of GR (a normal mechanism of negative feedback). This can be tested using the dexamethasone suppression test, and results consistently show an enhanced negative feedback response across many different populations with PTSD (240) and in animals exposed to SPS (90, 122, 241). One potential mechanism for this altered HPA axis activity is altered function and/or expression of steroid receptors, specifically GR and MR. The expression of GR and MR is programmed by stress and determines the responsiveness of neurons to circulating CORT; a high GR/MR ratio leads to increased sensitivity to the stress-related effects of increases in circulating CORT, including negative feedback of the HPA axis (242, 243). A large and growing body of evidence implicates altered expression and sensitivity of GR and MR in PTSD (88), and these targets, particularly an increased GR/MR ratio, may serve as useful biomarkers for PTSD phenotypes (36). Many studies have used the SPS model to investigate changes in HPA axis function and brain expression of GR and MR and to link these neurobiological changes to aberrant post-stress behaviors.

Mirroring clinical findings, SPS enhances rapid negative feedback of corticosterone (CORT; analogous to cortisol in humans) release (122, 138, 183, 241), and we have recently replicated this finding in mice (90). SPS also increases GR expression in many regions important for emotion, arousal, and memory, including HC (112, 184, 244), PFC (112, 245), and LC (246). Interestingly, no changes in GR as a result of SPS have been observed in AMY (184, 247). The effects of SPS on MR levels appear to be more complex. MR has been shown to decrease after SPS in HC (248) and LC (246), and MR expression in the mPFC appears to be biphasic, increasingly shortly after SPS, decreasing over the next 14 days, and then rebounding toward baseline (249).

Together these findings suggest that altered GR/MR expression may contribute to altered neuroendocrine response following traumatic stress and that this may underlie the phenotype seen following SPS. Supporting this, GR antagonists block SPS-enhanced contextual fear (122). Increased glucocorticoid signaling may contribute to PTSD-related memory impairment by promoting over-consolidation of aversive memories (250) and contributing to hypervigilance, anxiety, and emotional distress (251). This makes steroid receptor expression in the HPA axis and limbic brain regions a promising target for further investigation using the SPS model.

### Neurotransmitter and neuropeptide systems

#### Excitatory and inhibitory neurotransmission

Several behavioral domains disrupted by PTSD depend on the balance of inhibitory (e.g., glycine, GLY, and gamma-aminobutyric acid, GABA) and excitatory (e.g., glutamate; GLU) neurotransmission, including dissociation (252), sleep (253), and fear learning and extinction (254, 255); therefore, disruptions in these excitatory and inhibitory systems may underlie many of the behavioral changes seen in PTSD. Approaches using ^1^H-MRS to probe the GLU and GABA systems *in vivo* have identified differences between individuals with PTSD and controls. Notably, PTSD is associated with increased GABA (256) and decreased GLU (257) in ACC, which may predict hyperarousal symptoms (253). In contrast, temporal, parietal, occipital, and insular cortices tend to show decreased GABA and increased GLU in PTSD, and peripheral measures suggest an overall decrease in GABA and an overall increase in GLU (258). In addition, a PET study showed that GABA_A_ binding is decreased in the cortex, HC, and thalamus (259), indicating that PTSD also dysregulates GABA neurotransmission at the level of GABA receptors. These studies demonstrate that PTSD is associated with region-specific changes in excitatory and inhibitory neurotransmission which may contribute to PTSD symptomatology, making these systems important targets of pre-clinical study.

Several studies using SPS have investigated markers of GLU neurotransmission. SPS decreases mPFC, but not HC or AMY, GLU and glutamine 7 d after exposure (154, 260) and has also been observed to increase striatal expression of PSD-95, a glutamatergic synaptic marker (215). Additionally, increased phosphorylation of type 1A AMPA receptors in mPFC has been associated with cognitive inflexibility following SPS (151). In addition to these changes in mPFC GLU function, GLU levels in cerebral spinal fluid (CSF) increased 14 d after SPS exposure (130), suggesting brain-wide changes in GLU-based excitatory neurotransmission or metabolism. Finally, there is evidence that some drugs block the behavioral effects of SPS by regulating GLU neurotransmission. For example, the HDAC inhibitor vorinostat blocked SPS-induced impairments in fear extinction and increased expression of type 2B NMDA receptors (261), and the anticonvulsant and AMPA receptor antagonist topiramate attenuated SPS-induced exaggeration of acoustic startle response (140). This evidence suggests that altered GLU signaling may be driving SPS-induced memory impairments.

Less is known about the effects of SPS on inhibitory neurotransmission. While one report showed decreased GABA levels in HC after SPS (198), another report found no changes in GABA in HC, AMY, or mPFC (154). HC glycine transporter was persistently increased following contextual fear conditioning in SPS-exposed, but not control, rats (121), and this upregulation was associated with decreased extracellular GLY (262). How this may contribute to PTSD-associated behavior is unclear and likely complex, especially as GLY has a role in regulating excitatory neurotransmission. Nonetheless, this system may be a useful target for treatment. For example, D-cycloserine, a partial agonist of GLY binding sites on NMDA receptors which can ameliorate SPS-induced impairments in fear extinction and alter GLU receptor expression in the HC (117), has been proposed as a potential therapeutic for treating anxiety and PTSD in humans (263). More research is necessary to determine how the closely-connected systems of GLU, GABA, and glycine signaling are related to maladaptive post-traumatic behaviors. Notably, GLU and CORT signaling converge onto intracellular pathways that are altered following SPS, suggesting that the interactions between GLU and CORT may be involved in some post-traumatic adaptations (264).

#### Neuromodulators

The monoamines, serotonin (5-hydroxytryptamine; 5-HT), dopamine (DA), and norepinephrine (NE), likely play a significant role in the neurobiology of PTSD (265). Indeed, the only FDA-approved medications for PTSD, sertraline (Zoloft) and paroxetine (Paxil), are selective 5-HT reuptake inhibitors (SSRIs). However, these drugs have only moderate efficacy (3), underscoring the need to better understand the contribution of monoamines to PTSD symptomatology and neuropathology. Much attention has been paid to 5-HT in PTSD research as it is the main target for SSRIs using in the treatment of PTSD.

The main source of 5-HT in the forebrain is the dorsal raphe nuclei. Serotoninergic projections are distributed widely throughout the brain, play roles in many behaviors including emotional, social, and reward-related behaviors, and likely interact with CRF and CORT signaling to regulate stress coping behavior (266) and contribute to stress-related disorders (267). As SSRIs improve some PTSD symptoms (3), dysregulated 5-HT release or signaling may underlie post-traumatic behavioral changes. Our own and others' findings show that the behavioral effects of SPS can be ameliorated by a variety of SSRIs, including escitalopram (114), fluoxetine (268), and paroxetine (90, 127, 269). SPS also dysregulates 5-HT receptor expression and signaling across diverse brain regions. For example, SPS increases expression of 5-HT2C receptors in the AMY (119), where these receptors regulate contextual fear and responses to anxiogenic stimuli (270), and increases 5HT1A receptors in the oculomotor nucleus (271). In contrast, SPS decreases HC expression of 5-HT3A receptors, which are thought to be involved in neurogenesis and antidepressant effects (272). This change has been indirectly linked to PTSD symptomology by the finding that an intrahippocampal 5-HT3A receptor agonist infused immediately after SPS blocked fear extinction deficits (181). SPS might also affect 5-HT release and/or reuptake, as 5-HT concentrations are increased in the HC following SPS (183) and 5-HT reuptake transporter (SERT) knockdown in dorsal raphe projection neurons prevented SPS-induced fear extinction deficits (182). Alterations in 5-HT have been linked to behavioral changes seen following SPS; for example, markers of 5-HT turnover in the dorsal HC has been correlated with increased fear generalization in mice exposed to SPS (30). Changes in 5-HT appear to be region-specific, as SPS has been found to cause no change in 5-HT release in the IL, although this structure regulates fear extinction and retention of extinguished fear response (114).

NE is released into the forebrain from the LC and plays a critical regulatory role in arousal and attention (155). Its dysfunction likely contributes to PTSD symptoms, particularly those involving perturbations of arousal such as hypervigilance, startle, sleep disruptions, and trauma-cue reactivity (273). Notably, individuals with PTSD show increased CSF concentrations of NE (274), suggesting increased NE release, and NE dysfunction predicts specific PTSD symptoms, such as disrupted sleep (275) and startle (276). Correspondingly, NE function appears to be disrupted by SPS. SPS decreases NE release in the IL (114) and increases tyrosine hydroxylase and dopamine β-hydroxylase levels, enzymes responsible for the synthesis of catecholamines, in the LC (277). Additionally, SPS decreases tonic firing but increases phasic firing in LC neurons (153), which may contribute to a hypervigilant state (155) contributing to the enhanced startle and fear extinction deficits seen following SPS. To date, no study has investigated this causal relationship; therefore, LC function and NE signaling are promising areas for further investigation and potential development of novel treatment targets.

DA is broadly implicated in a variety of adaptive functions, including movement, learning, emotion, reward, and motivation (278). Its dysregulation has been implicated in multiple psychiatric disorders including schizophrenia (279), depression (280), and addiction (281). Recent evidence suggests that DA might play a unique role in behaviors associated with PTSD, including fear learning and extinction (282), addiction (283), and anhedonia (74). Multiple clinical findings link DA dysfunction to PTSD: DA metabolites in CSF decrease following traumatic reminders (284), striatal DA transporter (DAT)expression is increased in PTSD patients (285), and polymorphisms in genes encoding DAT and DA receptors are associated with PTSD (286). Mirroring these findings, we have observed SPS-related decreases in DA levels and turnover (i.e., DOPAC/DA) (190), D2 receptor availability (190) and concentration (215), corresponding with increased DAT concentrations in the striatum (190). SPS also decreases DA release in the PFC (114), which may promote extinction learning deficits (58). These effects may also be related to changes in reward function, affect, and sensitization to drugs of abuse described previously. Thus, future studies into the mechanisms for these changes in monoamines by SPS has the potential to provide great insight into affective and motivational symptoms of PTSD, including the increased risk of substance use in PTSD (287), and facilitate the development of targeted therapeutics.

Finally, acetylcholine (ACh), which signals through a variety of receptor subtypes to regulate both excitatory and inhibitory neurotransmission centrally and in the parasympathetic nervous system, may contribute to SPS phenotypes. Initial evidence, though scant, indicates that SPS increases muscarinic ACh receptor binding in HC and mPFC (132) and also that activation of spinal α7 nicotinic ACh receptors can reverse SPS-induced hyperalgesia (288). Especially in light of clinical findings that PTSD symptoms are associated with parasympathetic dysregulation (289) this suggests that the ACh system may be a promising target for treating both the central and peripheral pathophysiology of PTSD.

#### Cannabinoids

Cannabinoids (CBs) are lipid-based neuromodulators that largely inhibit neural function throughout the brain by acting as a retrograde neurotransmitter (290). The CB system appears to be altered in PTSD, and the recent, rapid development of CB research and pharmacology makes this a potentially fertile field for new interventions for PTSD (291). For example, THC (292) and the synthetic CB nabilone (293, 294) have been investigated in early clinical trials for PTSD-related nightmares and sleep disruption, and oral THC can facilitate successful recall of extinction memory in humans (295). Pre-clinical research suggests a role for CB signaling in other symptoms of PTSD, informed by the common use of cannabis as a coping behavior following the onset of PTSD (160, 296). The CB receptor agonist, Win55,212-2, can block SPS-induced impairment of contextual fear extinction when administered systemically or infused directly into BLA, subiculum, or vmPFC (116, 138). This effect appears to rely on an interaction with GR signaling, as GR blockade in the subiculum or BLA eliminates this effect (116). This is in line with evidence indicating that CB receptors in BLA modulate stress effects on affective memories (297). Additionally, mice exposed to SPS followed by ethanol administration have decreased CB1 receptor expression in the striatum (215). Further investigation of CB mechanisms using the SPS model could help explain the widespread use and abuse of cannabis in PTSD patients, as well as yield additional targeted treatments for PTSD.

#### Neuropeptides

A variety of neuropeptides have been implicated in stress responses and many are known to regulate HPA axis function. We focus here on the neuropeptides that have been shown to be affected *both* in PTSD and SPS, including oxytocin and NPY.

Oxytocin has important roles in social behavior and emotional reactivity (298). Variation in oxytocin/vasopressin genes might be related to the risk of PTSD development (299, 300), and oxytocin may facilitate effective psychotherapy for PTSD (301) and even prevent the development of PTSD in some individuals (302). Studies using SPS have corroborated these findings, showing SPS-induced increases in oxytocin receptor (OXTR) availability in the AMY, hypothalamus, and HC (89). Despite these findings, oxytocin administration does not reverse SPS-induced deficits in extinction learning (115). Nonetheless, a recent clinical trial found that oxytocin had significant effects in preventing the development of PTSD in a subgroup of patients showing high acute symptoms when given shortly following trauma (303). Further work to evaluate the mechanisms by which SPS affects oxytocin signaling may be fruitful in determining the potential of the oxytocin system as a treatment target for PTSD.

NPY is widely distributed in the brain and plays a role in fear (304), arousal (305), and traumatic stress responses (306). Clinical studies have linked variations in plasma NPY and polymorphisms in genes for NPY and its receptors with PTSD (307), making this system an attractive pharmacotherapeutic target for stress-related disorders (306). Several studies using SPS indicate that NPY interacts with brain monoamine systems and the HPA axis to orchestrate post-SPS behavioral changes. SPS decreases NPY receptor subtype 2, while at the same time increasing CRH and CRH receptor subtype 1 expression in the LC (277). Intranasal NPY administration shortly after SPS can prevent these changes as well as rescue SPS-induced increases in hippocampal GR and SPS-induced behavioral abnormalities (139, 178, 308). SPS also increases the number of NPY-reactive fibers in the BLA, which are positioned in contact with GR/MR-containing pyramidal neurons; this change is associated with an increase in morphological complexity in BLA neurons, but it is as yet unclear what role NPY plays in this change (247). These studies suggest that the role of the NPY system in regulating post-SPS changes in neurophysiology and behavior may be a promising field of study using the SPS model.

### Cellular adaptations in SPS

A unique advantage of pre-clinical models is that they allow insights into cellular processes underlying disease development and progression. Recent studies have begun to interrogate the effects of SPS on cellular and intracellular adaptations in the brain. Neuronal morphology can adapt in response to various stimuli, including stress (309), and determines the functional properties of neurons and their synaptic connections (310, 311). Studies of neural morphological changes following SPS have identified increased dendritic branching in the AMY (247) which may be related to altered fear conditioning, and somatic changes suggesting neuronal damage and apoptosis in the AMY (122) and HC (312, 313). Studies using SPS have not yet replicated findings from other pre-clinical models of traumatic stress showing reduced dendritic branching in the HC (314), which may explain the observation in rodents (315) and humans (45, 46) that HC volume is decreased following traumatic stress. While expanding and clarifying research on the effects of SPS on neuronal morphology remains an important goal, several laboratories have developed research programs aimed at unraveling the intracellular mechanisms that regulate responses to SPS, with a focus on mitogen activated-protein kinase (MAPK), protein kinase B (also known as Akt), and apoptotic signaling pathways.

#### Signal transduction pathways

Signaling from G-protein coupled receptors (including many neuropeptide receptors, muscarinic receptors, and nearly all monoamine receptors) is propagated through multiple intracellular signaling pathways, including the MAPK pathway (316) and the PI3K/Akt/mTOR pathway (317). The MAPK pathway responds to a wide variety of extracellular signals to regulate many cellular processes such as differentiation, growth, and apoptosis (318). In neurons, the MAPK pathway appears to be an important regulator of neuroplasticity (319). Early investigations into the effects of SPS on the MAPK pathway found that SPS increases the phosphorylation (activation) of MAPK in the mPFC (171, 320, 321) and AMY (147, 322, 323). These studies suggest that MAPK signaling plays a role in coordinating transcriptional responses to SPS via activation of cFos (320), in regulating neuronal apoptosis (323), and in the behavioral effects of SPS including hyperalgesia (171) and increased anxiety-like behavior (147).

The PI3K/Akt/mTOR pathway is activated by a wide variety of receptors, and coordinates cellular responses such as cell growth, proliferation, and survival via signaling through the serine/threonine protein kinases Akt and mTOR (317). We reported that SPS increased levels of phosphorylation (activation) of Akt in the HC concurrently with increases in GR (184). Increased Akt phosphorylation has been linked to GR activation (324) which may contribute to cell death (325). While this coincides with research using other stress models that suggest that Akt/mTOR activation is involved in responses to traumatic stress (326–328) another group has reported phasic decreases in Akt and mTOR phosphorylation following SPS (329). Further research is required to resolve this discrepancy and clarify the role of the PI3K/Akt/mTOR signaling pathway in adaptations following traumatic stress.

Both the MAPK and PI3K/Akt/mTOR pathways are involved in regulating synaptic plasticity and cellular morphology, particularly changes underlying fear learning (255). While relatively few studies using SPS have examined these effects, there is some indication that SPS produces morphological changes in neurons, specifically effects on soma size and dendrites. SPS has been observed to induce dendritic arborization in the BLA (247), changes which resemble changes in HC morphology observed following predator scent exposure, another traumatic stress model (314). Dendritic arborization may underlie neuronal plasticity and is controlled by diverse processes, including CORT response (330). Soma size and dendritic density were increased following SPS in vasopressin neurons in the hypothalamus, and these neurons had a blunted morphological response to an acute stressor (331). Neurotrophic signaling (e.g., through brain-derived neurotropic factor; BDNF) (332) has also been found to be altered by SPS, although results are inconsistent: some studies have shown that BDNF and components of its signaling pathway were upregulated following SPS (128, 147) while in another SPS appeared to decrease BDNF mRNA (168). There is also direct evidence that synaptic plasticity is altered by SPS. Specifically, hippocampal LTD is enhanced 1 day after SPS, and LTP in the HC and AMY is impaired 7 day after SPS (122). Additionally, SPS impacts cellular morphology. These findings suggest that changes in cellular physiology may contribute to the symptoms of PTSD, but do not provide a direct link between changes in signaling factors, cellular function, and behavioral changes. Further work is necessary to address this research gap, which will allow the investigation of interventions to prevent or reverse such changes in cellular plasticity following traumatic stress exposure.

#### Apoptotic pathways

In addition to changes in cellular morphology and electrophysiology, several studies have reported changes in histological and biochemical markers of apoptosis in brain tissue following SPS. Post-SPS apoptosis has been reported in up to 20% of AMY neurons (194, 333), in up to 45% of LC neurons (334), and in up to 20% of HC neurons (335). Multiple mechanisms to account for this observed loss of neurons have been proposed; these include oxidative-stress-induced loss of interneurons (123) and mitochondria-mediated apoptosis in the HC (313), multiple endoplasmic reticulum-based signaling pathways in the mPFC (321, 336), and caspase activation in the AMY (337). Some results disagree with these. For example, one study investigating changes in HC following SPS revealed no signs of neuronal death or morphological changes (122). Additionally, studies on post-SPS apoptosis so far do not routinely include measurements in brain areas that are not expected to show changes based on the specific pattern of post-SPS behavioral changes. If SPS (and potentially traumatic stress in general) causes apoptosis specifically in areas related to fear, anxiety, and stress-related behaviors, this may account for many behavioral changes seen in PTSD. On the other hand, these data may indicate that SPS produces widespread, non-specific apoptosis, which is unlikely to be mirrored in the human condition. While it is presently impossible to directly assess apoptosis in living patients with PTSD, post-mortem PFC samples from PTSD patients show dysregulation of cell survival and apoptosis-related genes (338), suggesting that apoptosis is abnormal in some way in people with PTSD. Stress-induced apoptosis may underlie differences in hippocampal volume seen in PTSD, but other phenomena could be at play; a study using mice showed that SPS causes HC volume loss along with a decrease in markers of axonal growth, indicating that reduced axonal size may underlie the change in HC size rather than changes in cell number or soma size (315). Further research on cell cycle regulation, neurogenesis, and apoptosis following SPS is important in resolving whether neuronal apoptosis following SPS is regionally specific or widespread, what its role in post-SPS behavioral changes is, and how it relates to human neurobiology.

### Inflammation and glia

Neuroinflammation has been proposed to play a part in a diverse set of psychiatric conditions, including major depressive disorder, bipolar disorder, schizophrenia, and PTSD (339, 340). Results from studies investigating inflammatory biomarkers have found that C-reactive protein and NF-kB pathway components are related to PTSD, and can even predict symptom severity (341–343). Inflammation may be related to the temporal course of PTSD, as inflammatory markers are only increased in those with current PTSD and return to baseline in individuals who have recovered (344). This is consistent with a wide body of research showing that GR signaling, which is altered in PTSD and following SPS, regulates immune function (345).

Following SPS, inflammation appears to be increased in the HC. SPS causes a consistent increase in IL-6 (123, 346) and a less consistent effect on TNFα, with one study showing increased TNFα after SPS (168) and another showing no change (123). An “enhanced SPS” protocol that includes an inescapable foot shock has been shown to increase IL-6, IL-2B, and iNOS levels in the HC, and these changes can be mitigated by administration of the NOS and COX-2 inhibitor gastrodin (346). Further supporting the hypothesis that inflammation plays a role in post-SPS behavioral changes, a 14-day treatment of the non-steroidal anti-inflammatory drug ibuprofen reversed SPS-induced increases in circulating CORT, anxiety-like behavior, and HC markers of inflammation (168).

There is also evidence that glial processes may contribute to post-SPS dysfunction and poor behavioral recovery. For example, microglial activation caused by glucocorticoid signaling in the spinal cord (288, 347) and HC (348) appears to contribute to post-SPS hyperalgesia. Immunohistochemical data indicate that glial cell number is decreased in the HC in a time-dependent manner following SPS, and magnetic resonance spectroscopy findings of decreased choline in the HC (312) and creatine in the HC (312) and mPFC (154) are consistent with a glial-related mechanism (312). Especially given the clinical association between PTSD and inflammatory disease, further research on the effects of SPS on inflammatory mediators and their relationship to post-SPS behavioral aberrations is needed in our understanding of the pathogenesis of PTSD.

Glia may participate in non-inflammatory changes following SPS, as well. Astroglia regulate processes important to neuronal function including synapse formation and GLU processing (349) and exhibit changes following other laboratory stressors (350). A post-SPS loss of cells expressing glial fibrillary acidic protein in the HC suggests a decrease in astroglial number(312), while administration of fibroblast growth factor 2, which regulates glial differentiation, has been found to mitigate the effects of SPS on contextual fear conditioning and unconditioned anxiety, possibly by restoring altered astrocytic GLU processing (130, 351). As astroglia actively regulate neuronal signaling, further research aimed at understanding changes in astroglial number, structure, and function following SPS may uncover mechanisms by which SPS alters neurotransmission.

It is unlikely that the intracellular factors heretofore investigated represent all factors involved in post-SPS adaptations. Discovery-focused approaches such as genomic and proteomic analyses have the potential to identify new molecules important in post-SPS adaptations. To date, only two studies report large-scale analyses of gene expression changes following SPS, including in the HC (119, 121) and ACC (119). While one study (121) found a set of differentially-expressed genes that were related to neurotransmission, the other (119) found that a diverse set of genes were affected, and the sets of genes reported to be differentially expressed following SPS in these two studies were non-overlapping. Further discovery-oriented research is necessary to clarify these results and identify other factors that may be involved in brain responses to traumatic stress.

## Conclusion

The SPS model of exposure to traumatic stress demonstrates significant validity as an animal model of PTSD. Its content validity is demonstrated by its ability to produce a behavioral and neurohormonal syndrome that corresponds to many characteristics of PTSD, particularly reactivity to trauma cues, impairment of fear extinction and extinction retention, hyperarousal, changes in neurotransmitter and neuropeptide systems, and altered responsivity of the HPA axis. Its criterion validity is demonstrated by the efficacy of many clinically utilized drugs in ameliorating post-SPS behavioral changes, and in the general agreement of findings in SPS with findings from other traumatic stress models and human laboratory research.

There are of course limits to the scope and validity of the SPS model. Like all animal models, it cannot be used straightforwardly to answer hypotheses about experiential (rather than behavioral) symptoms such as nightmares, intrusive thoughts, or trauma-related guilt. Although it is a complex, multimodal stressor, SPS does not account for many of the complex features of trauma experienced by humans that may influence the development of PTSD, including varying characteristics of traumatic events, historical and social context, and post-trauma cognitive and emotional processing. Species-level differences between rodents and humans in neuroanatomy and the neurobiology of stress must be considered when translating findings from model to clinic. Additionally, there are many things that can be measured in laboratory animals that are unlikely to ever be corroborated with data from human patients; for example, invasive measurements of brain function and measurements taken immediately pre- and post-trauma are difficult, if not impossible, to obtain in humans. Like pre-clinical research on traumatic stress as a whole, research using the SPS model has not yet led to the development of new treatments for PTSD, and so it has not yet achieved the strongest demonstration of predictive validity. Finally, SPS (like any models of traumatic stress) is likely to correspond closely to only a subset of individuals with PTSD—particularly those whose trauma was isolated to a single event—and may not correspond closely to adaptations seen in individuals who have survived multiple traumatic events, or particularly complex traumas such as those involving interpersonal interactions or bodily injury.

Despite these limitations, the SPS model stands out as a well-characterized, translationally tractable paradigm to study of the neurobiology of traumatic stress-induced adaptations in brain and behavior. In concert with other pre-clinical and clinical approaches to the study of traumatic stress and PTSD, SPS holds great potential to expand both our basic knowledge of the mechanisms by which traumatic stress affects behavior and the clinical armamentarium with which we can help individuals with PTSD achieve a greater quality of life.

## Author contributions

ML conducted the literature review and wrote the initial draft of this manuscript. AE, AC, IL, and SP advised ML in this process and provided revisions and critical feedback.

### Conflict of interest statement

The authors declare that the research was conducted in the absence of any commercial or financial relationships that could be construed as a potential conflict of interest.

## Abbreviations

5-HT: 5-hydroxytryptamine (serotonin)
ACC: anterior cingulate
ACh: acetylcholine
ACTH: adrenocorticotropic hormone
Akt: protein kinase B
AMY: amygdala
AMPA: α-amino-3-hydroxy-5-methyl-4-isoxazolepropionic acid
CB1: cannabinoid receptor, subtype 1
CeA: central amygdala
CSF: cerebrospinal fluid
CORT: corticosterone/cortisol
COX-2: cyclooxygenase-2
CRH: corticotropin releasing hormone
D2, dopamine receptor: subtype 2
DA: dopamine
DAT: dopamine transporter
DSM-5: Diagnostic and Statistical Manual of Mental Disorders, Fifth Edition
FDA: US Food and Drug Administration
GABA: gamma-aminobutyric acid
GAP43: growth associated protein 43
GLU: glutamate
GLY: glycine
GR: glucocorticoid receptor
HC: hippocampus
HPA: hypothalamus-pituitary-adrenal
IL-2B: interleukin 2B
IL-6: interleukin 6
ILC: infralimbic cortex
iNOS: calcium-insensitive nitic oxide synthase
LC: locus coeruleus
LTP: long-term potentiation
MAPK: mitogen activated protein kinase
mPFC: medial prefrontal cortex
MR: mineralocorticoid receptor
mTOR: mammalian target of rapamycin
NE: norepinephrine
NF-κB: nuclear factor κ-light-chain-enhancer of activated B cells
NOS: nitric oxide synthase
NPY: neuropeptide Y
PFC: prefrontal cortex
PI3K: phosphatidylinositide 3-kinase
PKC: protein kinase C
PSD-95: post-synaptic density 95
PTSD: post-traumatic stress disorder
SPS: single prolonged stress
SERT: 5-HT transporter
SSRI: selective serotonin reuptake inhibitor
THC: Tetrahydrocannabinol
TNFα: tumor necrosis factor α
vmPFC: ventromedial prefrontal cortex.
