# Supplementary material for: Single-Prolonged Stress: A Review of Two Decades of Progress in a Rodent Model of Post-traumatic Stress Disorder
Source: Front Psychiatry. 2018 May 15;9:196. doi: 10.3389/fpsyt.2018.00196 (PMC5962709; doi:10.3389/fpsyt.2018.00196)
Supplement: Supplementary file 1 [file Data_Sheet_1.docx]

Supplementary Table 1

Summary of observed behavioral effects of SPS exposure.

| **RDoC Construct** | **Behavior** | **Most Replicated Finding** | **Other Findings** |
| --- | --- | --- | --- |
| **Acute Threat (“Fear”)** | Recall of fear conditioning | ↑ (1-19) | No effect (20-25) |
|  | Extinction of fear conditioning | ↓ (20, 24, 26, 27) | No effect (21-23, 25, 28) |
|  | Retention of fear extinction | ↓ (21, 23-25, 27-32) |  |
|  | Generalization of fear to CS- | ↑ (13, 17, 33) |  |
|  | Fear response to trauma-associated cues | ↑ (18, 28, 34-36) |  |
|  | Learned helplessness* | ↑ (37-42) |  |
| **Negative Valence Systems - Potential Threat (“Anxiety”)** | Thigmotaxis in open field | ↑ (3, 13, 19, 40-52) | No effect (53) |
|  | Anxiety-like behavior in elevated plus maze | ↑ (1-4, 8, 11-13, 15, 17, 18, 35, 37-41, 43, 44, 46, 48-50, 52, 54-63) | No effect (27, 53, 64, 65) |
|  | Avoidance in light-dark box | ↑ (18, 36, 51, 54, 55) | No effect (27, 64, 65) |
| **Arousal** | Startle | ↑ (9, 51, 66, 67) | ↓ (18, 34-36) |
|  | Sensory reactivity** | ↑ (56, 57, 62, 68-72) | ↓ (8, 10) No effect (9) |
|  | Motor Activity | No effect (1, 2, 12, 13, 17, 19, 21, 39, 41, 52, 73) | ↓ (18, 42, 74, 75) |
| **Circadian Rhythms** | REM sleep | ↑ (32, 76, 77) | ↓ (78) |
|  | Total sleep | ↓ (78) |  |
| **Approach Motivation - Reward Valuation** | Consumption of food reward | ↓ (25, 47, 79) |  |
| **Reward Learning** | Cocaine self-administration | ↓ (79-81) | No effect (82) |
|  | Cocaine place preference | ↓ (79) |  |
|  | Ethanol place preference | ↑ (11) |  |
| **Habit** | Cocaine sensitization | ↑ (82) |  |
|  | Methamphetamine sensitization | ↑ (83) |  |
|  | Amphetamine sensitization | ↓ (34) |  |
|  | Ethanol sensitization | ↓ (74) |  |
| **Declarative Memory** | Spatial Memory | ↓ (9, 45, 47, 52, 58, 61, 62, 71, 84-88) |  |
|  | Recognition | ↓ (3, 55, 85, 89) |  |
| **Performance Monitoring** | Set Shifting and Reversal Learning | ↓ (90, 91) |  |
| **Affiliation** | Investigation of conspecifics | ↓ (89) |  |

* Learned helplessness (seen in the forced swim test) is included here as a behavior related to acute threat (“fear”), however, RDoC does not include it as a separate construct (92) it has also been describe as an abnormal suppression of escape from an aversive stimulus (93), aligning it with the response selection; inhibition/suppression construct in the domain of cognitive control.

** Most findings of increased sensory reactivity are related to pain or aversive stimuli; while pain is not currently a discrete concept in the RDoC system, it has been discussed as a possible distinct construct within the domain of “Negative Valence Systems” (94).

**Supplementary Table 2**

Summary of observed neurobiological effects of SPS exposure.

| **System** | | **Target** | **Region** | **Most replicated finding** | **Other findings** |
| --- | --- | --- | --- | --- | --- |
| **Neuroendocrine Processes** | **HPA axis** | Basal circulating Corticosterone | Serum | No effect (2, 9, 28, 34, 51, 95, 96) | ↑ (39, 46, 67, 71)  ↓ (21) |
|  |  | Basal circulating ACTH | Serum | No effect (9, 57, 96, 97) | ↑ (67, 71) |
|  |  | Dexamethasone suppression | Serum | ↑ (2, 9, 28, 51, 55, 57, 97, 98) |  |
|  |  | GR Expression | Amygdala | No effect (65, 67, 99, 100) | ↑ (101)  ↓ (59) |
|  |  |  | Hippocampus | ↑ (22, 29, 38, 65, 67, 100-102) | ↓ (103)  No effect (22, 104) |
|  |  |  | Locus Coeruleus | (105) |  |
|  |  |  | Medial prefrontal cortex | ↑ (22, 100, 101) | No effect (67) |
|  |  | MR expression | Hippocampus | ↓ (22, 98, 100, 103) | No effect (104) |
|  |  |  | Amygdala | ↓ (59) | No Effect (99) |
|  |  |  | Locus Coeruleus | ↓ (105) |  |
|  |  |  | Medial prefrontal cortex | ↓ (106) |  |
|  |  | CRF | Hypothalamus | ↑ (107) |  |
|  |  |  | Amygdala | ↑ (102) |  |
| **Neurotransmitter and Neuromodulator Systems** | **Glutamate** | Glutamate | Medial Prefrontal cortex | ↓ (28, 108, 109) |  |
|  |  |  | Amygdala | ↓ (109) |  |
|  |  |  | Hippocampus | ↓ (109) |  |
|  |  | NMDA Receptors | Amygdala | ↑ (20) |  |
|  |  |  | Hippocampus | No effect (110) | ↓ (111) |
|  |  | Glycine Transporter | Hippocampus | No effect (7) |  |
|  |  |  | Hippocampus; after contextual fear conditioning | ↑ (7) |  |
|  |  | VAMP2 | Hippocampus | No effect (7) |  |
|  |  |  | Hippocampus; after contextual fear conditioning | ↑ (7) |  |
|  |  | PSD95 | Striatum | ↑ (74) |  |
|  | **GABA** | GABA | Medial prefrontal cortex | No effect (109) |  |
|  |  |  | Amygdala | No effect (109) |  |
|  |  |  | Hippocampus | ↓ (111) | No effect (109) |
|  | **Norepinephrine** | Norepinephrine | Hippocampus | No effect (4, 21) | ↑ (112) |
|  |  |  | Medial prefrontal cortex | No effect (4, 112) | ↑ (21) |
|  |  |  | Amygdala | ↑ (21) |  |
|  |  | Dopamine beta hydroxylase | Basolateral amygdala | No effect (99) |  |
|  |  |  | Locus coeruleus | ↑ (107) |  |
|  |  | Tyrosine Hydroxylase | Locus coeruleus | ↑ (38, 67, 107, 113) |  |
|  |  | Stimulus-evoked response | Locus coeruleus | ↑ (113) |  |
|  |  | Norepinephrine Transporter | Medial prefrontal cortex | ↓ (21) |  |
|  |  |  | Amygdala | No effect (21) |  |
|  |  |  | Hippocampus | No effect (21) |  |
|  | **Serotonin** | Serotonin | Hippocampus | ↓ (4, 64) | ↑ (112), No effect (78) |
|  |  |  | Amygdala | No effect (33, 78) |  |
|  |  |  | Prefrontal cortex | No effect (4, 78, 112) |  |
|  |  | 5HT_2C_ Receptor | Amygdala | ↑ (114) |  |
|  |  | 5HT_1A_ Receptor | Dorsal Raphe | ↑ (115) |  |
|  |  |  | Oculomotor Nucleus | ↑ (116) |  |
|  |  | 5HT_3A_ Receptor | Hippocampus | ↓ (27) |  |
|  | **Dopamine** | Dopamine | Hippocampus | No effect (4, 21) | ↑ (112) |
|  |  |  | Nucleus Accumbens | ↓ (79) |  |
|  |  |  | Dorsal Striatum | ↓ (79) |  |
|  |  |  | Medial prefrontal cortex | ↑ (21, 112) |  |
|  |  |  | Amygdala | ↑ (21) |  |
|  |  | D1 Receptor | Nucleus Accumbens | No effect (79) |  |
|  |  |  | Caudate Putamen | No effect (79) |  |
|  |  | D2 Receptor | Nucleus Accumbens | ↓ (79) |  |
|  |  |  | Caudate Putamen | ↓ (79) |  |
|  |  | Dopamine Transporter | Nucleus Accumbens | ↑ (79) |  |
|  |  |  | Caudate Putamen | No effect (79) |  |
|  |  |  | Medial prefrontal cortex | No effect (21) |  |
|  |  |  | Amygdala | ↓ (21) |  |
|  |  |  | Hippocampus | ↓ (21) |  |
|  | **Neuropeptides** | Nociceptin/Orphanin FQ | Serum | ↑ (57) |  |
|  |  |  | CSF | ↑ (57) |  |
|  |  | NPY | Hippocampus | No effect (99) |  |
|  |  |  | Amygdala | ↑ (99) | ↓ (78) |
|  |  |  | Medial Prefrontal Cortex | No effect (99) |  |
|  |  |  | Hypothalamus | ↓ (46) | No effect (99) |
|  |  |  | Locus Coeruleus | ↑ (107) |  |
|  |  | NPY Receptors | Amygdala | No effect (78) |  |
|  |  | Arginine Vasopressin | Hypothalamus | ↑ (96) |  |
|  |  | Oxytocin Receptor | Hippocampus | ↑ (117) |  |
|  |  |  | Hypothalamus | No effect (117) |  |
|  |  |  | Amygdala | ↑ (117) |  |
|  | **GPCR Regulation** | Β-arrestin | Amygdala | ↓ (118) |  |
| **Cellular Adaptations** | **Neurogenesis Regulation** | BDNF | Hippocampus | ↑ (6, 55) | ↓ (119) |
|  |  |  | Amygdala | ↑ (52) |  |
|  |  |  | Serum | No effect (55) |  |
|  |  | TRK-B | Hippocampus | ↑ (6) |  |
|  |  | TRK-B phosphorylation | Amygdala | ↑ (52) |  |
|  |  | Neurogenesis | Hippocampus | ↓ (17, 49) |  |
|  |  | Progenitor cells | Subgranular zone | ↓ (64) |  |
|  |  |  |  |  |  |
|  | **Synaptic Plasticity** | LTP | Hippocampus | ↓ (86) |  |
| **Intracellular Signaling** | **Calcium regulation** | VDR | Hippocampus | ↑ (120) |  |
|  |  | LVSCC-A1C | Hippocampus | ↑ (120) |  |
|  |  | Free Ca++ | Hippocampus | ↑ (120, 121) |  |
|  |  |  | Amygdala | ↑ (122) |  |
|  |  |  | Medial prefrontal cortex | ↑ (87, 123, 124) |  |
|  |  | CRT | Medial prefrontal cortex | ↑ (87) |  |
|  | **Protein Kinase C pathway** | PKM(zeta) | Hippocampus | ↑ (41) |  |
|  |  | PKC(zeta) | Hippocampus | ↑ (41) |  |
|  |  | PKC(gamma) | Dorsal horn of spinal cord | ↑ (72) |  |
|  |  | PKC | Medial Prefrontal Cortex | ↑ (87) |  |
|  | **Protein Kinase A pathway** | PKA | Amygdala | ↑ (118) |  |
|  |  | PDE-4 | Amygdala | ↓ (118) |  |
|  |  | cAMP | Amygdala | ↑ (118) |  |
|  |  | CREB | Amygdala | ↑ (118) |  |
|  | **Calcium-activated mitogen kinase II pathway** | CAMKIIA | Hippocampus | ↓ (121, 125) | No effect (110) |
|  |  |  | Medial prefrontal cortex | ↓ (123) |  |
|  |  |  | Amygdala | ↓ (122) |  |
|  |  | CAMKIIB | Hippocampus | No Effect (110) |  |
|  |  | CAM | Hippocampus | ↑ (121, 125) |  |
|  |  |  | Medial prefrontal cortex | ↑ (123) |  |
|  |  |  | Amygdala | ↑ (122) |  |
|  |  |  | Dorsal raphe | ↑ (126) |  |
|  | **ERK Pathway** | ERK1/2 Phosphorylation | Hippocampus | ↓ (17) |  |
|  |  |  | Medial prefrontal cortex | ↑ (62, 127, 128) |  |
|  |  |  | Amygdala | ↑ (52, 129) |  |
|  |  | PERK | Medial prefrontal cortex | ↑ (85) |  |
|  |  | PERK phosphorylation | Medial Prefrontal Cortex | ↑ (85, 124) |  |
|  |  | P38 MAPK | Hippocampus | No effect (48) |  |
|  |  |  | Amygdala | ↑ (52) |  |
|  |  |  | Dorsal Raphe Nucleus | ↑ (84) |  |
|  |  | P38 MAPK phosphorylation | Hippocampus | ↑ (48) |  |
|  |  | AKT | Hippocampus | No effect (65) |  |
|  |  |  | Amygdala | No effect (65) |  |
|  |  | AKT phosphorylation | Hippocampus | ↑ (65) |  |
|  |  |  | Amygdala | No effect (65) |  |
|  |  | eIF2a | Medial Prefrontal Cortex | ↑ (85) |  |
|  |  | eIF2a phosphorylation | Medial Prefrontal Cortex | ↑ (85, 124) |  |
|  | **Apoptosis Pathways** | Apoptosis (Morphological evidence, DNA fragmentation) | Hippocampus | ↑ (3, 27, 43, 61, 86, 121, 125, 130-132) |  |
|  |  |  | Amygdala | ↑ (129, 133, 134) |  |
|  |  |  | Medial prefrontal cortex | ↑ (45, 85, 124, 135) |  |
|  |  |  | Locus Coeruleus | ↑ (136) |  |
|  |  |  | Dorsal Raphe Nucleus | ↑ (137) |  |
|  |  | Neuronal Density | Hippocampus | ↓ (16, 19, 61) |  |
|  |  | Bcl-2 | Hippocampus | ↑ (3, 55, 86, 132) |  |
|  |  |  | Amygdala | ↑ (129, 133, 138) |  |
|  |  |  | Medial Prefrontal Cortex | ↑ (129, 133, 138) (85) |  |
|  |  |  | Dorsal Raphe Nucleus | ↑ (84) |  |
|  |  | Caspases | Hippocampus | ↑ (55, 86, 125, 130) |  |
|  |  |  | Amygdala | ↑ (134, 139) |  |
|  |  |  | Medial Prefrontal Cortex | ↑ (45, 85, 88, 124, 140) |  |
|  |  | Unfolded Protein Response | Hippocampus | ↑ (125) |  |
|  |  |  | Amygdala | ↑ (134) |  |
|  |  |  | Medial prefrontal cortex | ↑ (45, 85, 88, 124) |  |
|  |  |  | Locus coeruleus | ↑ (136) |  |
|  |  |  | Dorsal Raphe Nucleus | ↑ (84, 141) |  |
|  |  | COX | Hippocampus | ↑ (132) |  |
|  |  |  | Amygdala | ↓ (139) |  |
|  |  | Beclin-1 | Hippocampus | ↑ (27, 64, 132) |  |
|  |  |  | Medial Prefrontal Cortex | ↑ (140) |  |
|  |  | JNK | Dorsal Raphe Nucleus | ↑ (84) |  |
|  |  | ASK1 | Dorsal Raphe Nucleus | ↑ (84) |  |
| **Glia and Immune Modulators** | **Immune Modulators** |  | Hippocampus | ↑ (19, 48, 56, 119) (56, 119) (48, 111) |  |
|  |  |  | Lumbar Spine | ↑ (48, 56, 69, 111) |  |
|  | **Glial Markers** | GFAP | Hippocampus | ↓ (44, 131) |  |
|  |  |  | Lumbar Spine | ↑ (69, 70) |  |

References

1. Qiu ZK, He JL, Liu X, Zeng J, Chen JS, Nie H. Anti-PTSD-like effects of albiflorin extracted from Radix paeoniae Alba. Journal of ethnopharmacology. 2017;198:324-30.

2. Miyake S, Koizumi S, Sasaguri K, Kawata T. Chewing ameliorates stress-induced enhancement of anxiety behaviours in an animal model of post-traumatic stress disorder. Biomedical Research. 2017.

3. Huang Z-l, Liu R, Bai X-y, Zhao G, Song J-k, Wu S, et al. Protective effects of the novel adenosine derivative WS0701 in a mouse model of posttraumatic stress disorder. Acta Pharmacologica Sinica. 2014;35(1):24-32.

4. Zhang L-M, Yao J-Z, Li Y, Li K, Chen H-X, Zhang Y-Z, et al. Anxiolytic effects of flavonoids in animal models of posttraumatic stress disorder. Evidence-Based Complementary and Alternative Medicine. 2012;2012.

5. Yamamoto S, Morinobu S, Iwamoto Y, Ueda Y, Takei S, Fujita Y, et al. Alterations in the hippocampal glycinergic system in an animal model of posttraumatic stress disorder. Journal of psychiatric research. 2010;44(15):1069-74.

6. Takei S, Morinobu S, Yamamoto S, Fuchikami M, Matsumoto T, Yamawaki S. Enhanced hippocampal BDNF/TrkB signaling in response to fear conditioning in an animal model of posttraumatic stress disorder. Journal of psychiatric research. 2011;45(4):460-8.

7. Iwamoto Y, Morinobu S, Takahashi T, Yamawaki S. Single prolonged stress increases contextual freezing and the expression of glycine transporter 1 and vesicle-associated membrane protein 2 mRNA in the hippocampus of rats. Progress in Neuro-Psychopharmacology and Biological Psychiatry. 2007;31(3):642-51.

8. Imanaka A, Morinobu S, Toki S, Yamawaki S. Importance of early environment in the development of post-traumatic stress disorder-like behaviors. Behavioural brain research. 2006;173(1):129-37.

9. Kohda K, Harada K, Kato K, Hoshino A, Motohashi J, Yamaji T, et al. Glucocorticoid receptor activation is involved in producing abnormal phenotypes of single-prolonged stress rats: a putative post-traumatic stress disorder model. Neuroscience. 2007;148(1):22-33.

10. Takahashi T, Morinobu S, Iwamoto Y, Yamawaki S. Effect of paroxetine on enhanced contextual fear induced by single prolonged stress in rats. Psychopharmacology. 2006;189(2):165-73.

11. Yu L, Wang L, Zhao X, Song M, Wang X. Role of single prolonged stress in acquisition of alcohol conditioned place preference in rats. Life sciences. 2016;151:259-63.

12. Miao Y-L, Guo W-Z, Shi W-Z, Fang W-W, Liu Y, Liu J, et al. Midazolam ameliorates the behavior deficits of a rat posttraumatic stress disorder model through dual 18 kDa translocator protein and central benzodiazepine receptor and neurosteroidogenesis. PloS one. 2014;9(7):e101450.

13. Feng D, Guo B, Liu G, Wang B, Wang W, Gao G, et al. FGF2 alleviates PTSD symptoms in rats by restoring GLAST function in astrocytes via the JAK/STAT pathway. European Neuropsychopharmacology. 2015;25(8):1287-99.

14. Mirshekar M, Abrari K, Goudarzi I, Rashidy-Pour A. Effects of β-Estradiol on Enhanced Conditioned Fear Induced by Single Prolonged Stress and Shock in Rats. Basic and Clinical Neuroscience. 2012;3(2):5-11.

15. Qiu Z-K, Zhang G-H, He J-L, Ma J-C, Zeng J, Shen D, et al. Free and Easy Wanderer Plus (FEWP) improves behavioral deficits in an animal model of post-traumatic stress disorder by stimulating allopregnanolone biosynthesis. Neuroscience letters. 2015;602:162-6.

16. Saffari S, Abrari K, Rezaei A, Rashidy-Pour A, Goudarzi I, Salmani ME. Correlation of fear memory in a PTSD animal model and hippocampal BDNF in response to β-estradiol treatment. Journal of Paramedical Sciences. 2015;6(3):22-34.

17. Nie H, Peng Z, Lao N, Wang H, Chen Y, Fang Z, et al. Rosmarinic acid ameliorates PTSD-like symptoms in a rat model and promotes cell proliferation in the hippocampus. Progress in Neuro-Psychopharmacology and Biological Psychiatry. 2014;51:16-22.

18. Le Dorze C, Gisquet-Verrier P. Sensitivity to trauma-associated cues is restricted to vulnerable traumatized rats and reinstated after extinction by yohimbine. Behavioural Brain Research. 2016;313:120-34.

19. Liu F-f, Yang L-d, Sun X-r, Zhang H, Pan W, Wang X-m, et al. NOX2 Mediated-Parvalbumin Interneuron Loss Might Contribute to Anxiety-Like and Enhanced Fear Learning Behavior in a Rat Model of Post-Traumatic Stress Disorder. Molecular neurobiology. 2015:1-10.

20. Yamamoto S, Morinobu S, Fuchikami M, Kurata A, Kozuru T, Yamawaki S. Effects of single prolonged stress and D-cycloserine on contextual fear extinction and hippocampal NMDA receptor expression in a rat model of PTSD. Neuropsychopharmacology. 2008;33(9):2108-16.

21. Lin C-C, Tung C-S, Lin P-H, Huang C-L, Liu Y-P. Traumatic stress causes distinctive effects on fear circuit catecholamines and the fear extinction profile in a rodent model of posttraumatic stress disorder. European Neuropsychopharmacology. 2016;26(9):1484-95.

22. George SA, Rodriguez-Santiago M, Riley J, Rodriguez E, Liberzon I. The effect of chronic phenytoin administration on single prolonged stress induced extinction retention deficits and glucocorticoid upregulation in the rat medial prefrontal cortex. Psychopharmacology. 2015;232(1):47-56.

23. Knox D, George SA, Fitzpatrick CJ, Rabinak CA, Maren S, Liberzon I. Single prolonged stress disrupts retention of extinguished fear in rats. Learning & Memory. 2012;19(2):43-9.

24. Knox D, Nault T, Henderson C, Liberzon I. Glucocorticoid receptors and extinction retention deficits in the single prolonged stress model. Neuroscience. 2012;223:163-73.

25. Lin C-C, Tung C-S, Liu Y-P. Escitalopram reversed the traumatic stress-induced depressed and anxiety-like symptoms but not the deficits of fear memory. Psychopharmacology. 2016;233(7):1135-46.

26. Wu ZM, Zheng CH, Zhu ZH, Wu FT, Ni GL, Liang Y. SiRNA-mediated serotonin transporter knockdown in the dorsal raphe nucleus rescues single prolonged stress-induced hippocampal autophagy in rats. J Neurol Sci. 2016;360:133-40.

27. Wu Z-M, Yang L-H, Cui R, Ni G-L, Wu F-T, Liang Y. Contribution of Hippocampal 5-HT3 Receptors in Hippocampal Autophagy and Extinction of Conditioned Fear Responses after a Single Prolonged Stress Exposure in Rats. Cellular and Molecular Neurobiology. 2016:1-12.

28. Perrine SA, Eagle AL, George SA, Mulo K, Kohler RJ, Gerard J, et al. Severe, multimodal stress exposure induces PTSD-like characteristics in a mouse model of single prolonged stress. Behavioural brain research. 2016;303:228-37.

29. Keller SM, Schreiber WB, Staib JM, Knox D. Sex differences in the single prolonged stress model. Behavioural brain research. 2015;286:29-32.

30. Keller SM, Schreiber WB, Stanfield BR, Knox D. Inhibiting corticosterone synthesis during fear memory formation exacerbates cued fear extinction memory deficits within the single prolonged stress model. Behavioural brain research. 2015;287:182-6.

31. Knox D, Stanfield BR, Staib JM, David NP, Keller SM, DePietro T. Neural circuits via which single prolonged stress exposure leads to fear extinction retention deficits. Learning & memory (Cold Spring Harbor, NY). 2016;23(12):689-98.

32. Vanderheyden WM, George SA, Urpa L, Kehoe M, Liberzon I, Poe GR. Sleep alterations following exposure to stress predict fear-associated memory impairments in a rodent model of PTSD. Experimental brain research. 2015;233(8):2335-46.

33. Aikins DE, Strader JA, Kohler RJ, Bihani N, Perrine SA. Differences in hippocampal serotonergic activity in a mouse single prolonged stress paradigm impact discriminant fear acquisition and retention. Neurosci Lett. 2017;639:162-6.

34. Toledano D, Tassin J-P, Gisquet-Verrier P. Traumatic stress in rats induces noradrenergic-dependent long-term behavioral sensitization: role of individual differences and similarities with dependence on drugs of abuse. Psychopharmacology. 2013;230(3):465-76.

35. Toledano D, Gisquet-Verrier P. Only susceptible rats exposed to a model of PTSD exhibit reactivity to trauma-related cues and other symptoms: An effect abolished by a single amphetamine injection. Behavioural brain research. 2014;272:165-74.

36. Le Dorze C, Gisquet-Verrier P. Effects of multiple brief exposures to trauma-associated cues on traumatized resilient and vulnerable rats. Brain Res. 2016;1652:71-80.

37. Serova LI, Laukova M, Alaluf LG, Sabban EL. Intranasal infusion of melanocortin receptor four (MC4R) antagonist to rats ameliorates development of depression and anxiety related symptoms induced by single prolonged stress. Behavioural brain research. 2013;250:139-47.

38. Sabban EL, Serova LI, Alaluf LG, Laukova M, Peddu C. Comparative effects of intranasal neuropeptide Y and HS014 in preventing anxiety and depressive-like behavior elicited by single prolonged stress. Behavioural brain research. 2015;295:9-16.

39. Lee B, Sur B, Yeom M, Shim I, Lee H, Hahm D-H. L-tetrahydropalmatine ameliorates development of anxiety and depression-related symptoms induced by single prolonged stress in rats. Biomolecules & therapeutics. 2014;22(3):213.

40. Solanki N, Alkadhi I, Atrooz F, Patki G, Salim S. Grape powder prevents cognitive, behavioral, and biochemical impairments in a rat model of posttraumatic stress disorder. Nutrition Research. 2015;35(1):65-75.

41. Ji L-L, Tong L, Xu B-K, Fu C-H, Shu W, Peng J-B, et al. Intra-hippocampal administration of ZIP alleviates depressive and anxiety-like responses in an animal model of posttraumatic stress disorder. Behavioral and brain functions. 2014;10(1):1.

42. Wu Z, Tian Q, Li F, Gao J, Liu Y, Mao M, et al. Behavioral changes over time in post-traumatic stress disorder: Insights from a rat model of single prolonged stress. Behavioural processes. 2016;124:123-9.

43. Zhao M, Yu Z, Zhang Y, Huang X, Hou J, Zhao Y, et al. Iron-induced neuronal damage in a rat model of post-traumatic stress disorder. Neuroscience. 2016.

44. Xia L, Zhai M, Wang L, Miao D, Zhu X, Wang W. FGF2 blocks PTSD symptoms via an astrocyte-based mechanism. Behavioural brain research. 2013;256:472-80.

45. Li X, Han F, Shi Y. IRE1α-XBP1 Pathway Is Activated Upon Induction of Single-Prolonged Stress in Rat Neurons of the Medial Prefrontal Cortex. Journal of Molecular Neuroscience. 2015;57(1):63-72.

46. Lee B, Sur B, Cho S-G, Yeom M, Shim I, Lee H, et al. Ginsenoside Rb1 rescues anxiety-like responses in a rat model of post-traumatic stress disorder. Journal of natural medicines. 2016;70(2):133-44.

47. Patki G, Li L, Allam F, Solanki N, Dao AT, Alkadhi K, et al. Moderate treadmill exercise rescues anxiety and depression-like behavior as well as memory impairment in a rat model of posttraumatic stress disorder. Physiology & behavior. 2014;130:47-53.

48. Peng Z, Wang H, Zhang R, Chen Y, Xue F, Nie H, et al. Gastrodin Ameliorates Anxiety-Like Behaviors and Inhibits IL-1 [Beta] Level and p38 MAPK Phosphorylation of Hippocampus in the Rat Model of Posttraumatic Stress Disorder. Physiological Research. 2013;62(5):537.

49. Peng Z, Zhang R, Wang H, Chen Y, Xue F, Wang L, et al. Ziprasidone ameliorates anxiety-like behaviors in a rat model of PTSD and up-regulates neurogenesis in the hippocampus and hippocampus-derived neural stem cells. Behavioural brain research. 2013;244:1-8.

50. Wang H, Peng Y, Tan Q, Chen Y, Zhang R, Qiao Y, et al. Quetiapine ameliorates anxiety-like behavior and cognitive impairments in stressed rats: implications for the treatment of posttraumatic stress disorder. Physiological Research. 2010;59(2):263.

51. Ganon-Elazar E, Akirav I. Cannabinoids prevent the development of behavioral and endocrine alterations in a rat model of intense stress. Neuropsychopharmacology. 2012;37(2):456-66.

52. Ji L-L, Peng J-B, Fu C-H, Cao D, Li D, Tong L, et al. Activation of Sigma-1 receptor ameliorates anxiety-like behavior and cognitive impairments in a rat model of post-traumatic stress disorder. Behavioural Brain Research. 2016;311(408-416).

53. Lisieski MJ, Perrine SA. Binge-pattern cocaine administration causes long-lasting behavioral hyperarousal but does not enhance vulnerability to single prolonged stress in rats. Psychiatry Research. 2017;257(Supplement C):95-101.

54. Masoumi-Ardakani Y, Mahmoudvand H, Mirzaei A, Esmaeilpour K, Ghazvini H, Khalifeh S, et al. The effect of Elettaria cardamomum extract on anxiety-like behavior in a rat model of post-traumatic stress disorder. Biomedicine & pharmacotherapy = Biomedecine & pharmacotherapie. 2017;87:489-95.

55. Shafia S, Vafaei AA, Samaei SA, Bandegi AR, Rafiei A, Valadan R, et al. Effects of moderate treadmill exercise and fluoxetine on behavioural and cognitive deficits, hypothalamic-pituitary-adrenal axis dysfunction and alternations in hippocampal BDNF and mRNA expression of apoptosis - related proteins in a rat model of post-traumatic stress disorder. Neurobiology of learning and memory. 2017;139:165-78.

56. Sun R, Zhang Z, Lei Y, Liu Y, Lu C, Rong H, et al. Hippocampal activation of microglia may underlie the shared neurobiology of comorbid posttraumatic stress disorder and chronic pain. Mol Pain. 2016;12.

57. Zhang Y, Gandhi PR, Standifer KM. Increased nociceptive sensitivity and nociceptin/orphanin FQ levels in a rat model of PTSD. Molecular pain. 2012;8(1):1.

58. Wang H-N, Peng Y, Tan Q-R, Wang H-H, Chen Y-C, Zhang R-G, et al. Free and Easy Wanderer Plus (FEWP), a polyherbal preparation, ameliorates PTSD-like behavior and cognitive impairments in stressed rats. Progress in Neuro-Psychopharmacology and Biological Psychiatry. 2009;33(8):1458-63.

59. Han F, Ding J, Shi Y. Expression of amygdala mineralocorticoid receptor and glucocorticoid receptor in the single-prolonged stress rats. BMC neuroscience. 2014;15(1):1.

60. Serova L, Laukova M, Alaluf L, Pucillo L, Sabban E. Intranasal neuropeptide Y reverses anxiety and depressive-like behavior impaired by single prolonged stress PTSD model. European Neuropsychopharmacology. 2014;24(1):142-7.

61. Peng Y, Feng S-F, Wang Q, Wang H-N, Hou W-G, Xiong L, et al. Hyperbaric oxygen preconditioning ameliorates anxiety-like behavior and cognitive impairments via upregulation of thioredoxin reductases in stressed rats. Progress in Neuro-Psychopharmacology and Biological Psychiatry. 2010;34(6):1018-25.

62. Qi J, Chen C, Lu Y-C, Zhang T, Xu H, Cui Y-Y, et al. Activation of extracellular signal-regulated kinase1/2 in the medial prefrontal cortex contributes to stress-induced hyperalgesia. Molecular neurobiology. 2014;50(3):1013-23.

63. Wang W, Liu Y, Zheng H, Wang HN, Jin X, Chen YC, et al. A modified single-prolonged stress model for post-traumatic stress disorder. Neuroscience letters. 2008;441(2):237-41.

64. Wu Z-M, Zheng C-H, Zhu Z-H, Wu F-T, Ni G-L, Liang Y. SiRNA-mediated serotonin transporter knockdown in the dorsal raphe nucleus rescues single prolonged stress-induced hippocampal autophagy in rats. Journal of the neurological sciences. 2016;360:133-40.

65. Eagle AL, Knox D, Roberts MM, Mulo K, Liberzon I, Galloway MP, et al. Single prolonged stress enhances hippocampal glucocorticoid receptor and phosphorylated protein kinase B levels. Neuroscience research. 2013;75(2):130-7.

66. Khan S, Liberzon I. Topiramate attenuates exaggerated acoustic startle in an animal model of PTSD. Psychopharmacology. 2004;172(2):225-9.

67. Serova L, Tillinger A, Alaluf L, Laukova M, Keegan K, Sabban E. Single intranasal neuropeptide Y infusion attenuates development of PTSD-like symptoms to traumatic stress in rats. Neuroscience. 2013;236:298-312.

68. Standifer KM, Simpson-Durand C, Zhang Y. Exacerbated Headache-Related Pain in the Single Prolonged Stress Model of Post-traumatic Stress Disorder. The FASEB Journal. 2016;30(1 Supplement):1265.8-.8.

69. Sun R, Zhang W, Bo J, Zhang Z, Lei Y, Huo W, et al. Spinal activation of alpha7-nicotinic acetylcholine receptor attenuates posttraumatic stress disorder-related chronic pain via suppression of glial activation. Neuroscience. 2017;344:243-54.

70. Qi J, Chen C, Meng QX, Wu Y, Wu H, Zhao TB. Crosstalk between Activated Microglia and Neurons in the Spinal Dorsal Horn Contributes to Stress-induced Hyperalgesia. Scientific reports. 2016;6:39442.

71. He YQ, Lang XQ, Lin L, Ji L, Yuan XY, Chen Q, et al. P2X3 receptor-mediated visceral hyperalgesia and neuronal sensitization following exposure to PTSD-like stress in the dorsal root ganglia of rats. Neurogastroenterol Motil. 2017;29(3).

72. He Y-Q, Chen Q, Ji L, Wang Z-G, Bai Z-H, Stephens RL, et al. PKCγ receptor mediates visceral nociception and hyperalgesia following exposure to PTSD-like stress in the spinal cord of rats. Molecular Pain. 2013;9(1):35.

73. Eskandarian S, Vafaei A, Vaezi GH, Taherian F, Kashefi A, Rashidy-Pour A. Effects of systemic administration of oxytocin on contextual fear extinction in a rat model of post-traumatic stress disorder. Basic and clinical neuroscience. 2013;4(4):315-22.

74. Matchynski-Franks JJ, Susick LL, Schneider BL, Perrine SA, Conti AC. Impaired Ethanol-Induced Sensitization and Decreased Cannabinoid Receptor-1 in a Model of Posttraumatic Stress Disorder. PloS one. 2016;11(5):e0155759.

75. Camp RL, Serova LI, Stier CL, Sabban EL. Effects of Intranasal NPY on Cardiovascular Parameters and Activity in SPS Model of PTSD: Telemetric Studies. The FASEB Journal. 2017;31(1 Supplement):1088.2-.2.

76. Vanderheyden W, Urpa L, Poe G. Increase in rem sleep following trauma exposure. Sleep Medicine. 2013;14:e293.

77. Vanderheyden WM, Poe GR, Liberzon I. Trauma exposure and sleep: using a rodent model to understand sleep function in PTSD. Experimental brain research. 2014;232(5):1575-84.

78. Nedelcovych MT, Gould RW, Zhan X, Bubser M, Gong X, Grannan M, et al. A Rodent Model of Traumatic Stress Induces Lasting Sleep and Quantitative Electroencephalographic Disturbances. ACS chemical neuroscience. 2015;6(3):485-93.

79. Enman NM, Arthur K, Ward SJ, Perrine SA, Unterwald EM. Anhedonia, reduced cocaine reward, and dopamine dysfunction in a rat model of posttraumatic stress disorder. Biological psychiatry. 2015;78(12):871-9.

80. Hofford RS, Prendergast MA, Bardo M. A modified single prolonged stress episode delays acquisition of cocaine self-administration. Drug & Alcohol Dependence. 2017;171:e90.

81. Hofford RS, Prendergast MA, Bardo MT. Modified single prolonged stress reduces cocaine self-administration during acquisition regardless of rearing environment. Behavioural Brain Research. 2018;338:143-52.

82. Eagle AL, Singh R, Kohler RJ, Friedman AL, Liebowitz CP, Galloway MP, et al. Single prolonged stress effects on sensitization to cocaine and cocaine self-administration in rats. Behavioural brain research. 2015;284:218-24.

83. Eagle AL, Perrine SA. Methamphetamine-induced behavioral sensitization in a rodent model of posttraumatic stress disorder. Drug and alcohol dependence. 2013;131(1):36-43.

84. Kong F, Han F, Xu Y, Shi Y. Molecular Mechanisms of IRE1alpha-ASK1 Pathway Reactions to Unfolded Protein Response in DRN Neurons of Post-Traumatic Stress Disorder Rats. Journal of molecular neuroscience : MN. 2017;61(4):531-41.

85. Wen L, Xiao B, Shi Y, Han F. PERK signalling pathway mediates single prolonged stress-induced dysfunction of medial prefrontal cortex neurons. Apoptosis : an international journal on programmed cell death. 2017;22(6):753-68.

86. Li X, Han F, Liu D, Shi Y. Changes of Bax, Bcl-2 and apoptosis in hippocampus in the rat model of post-traumatic stress disorder. Neurological research. 2010;32(6):579-86.

87. Wen L, Han F, Shi Y. Changes in the Glucocorticoid Receptor and Ca2+/Calreticulin-Dependent Signalling Pathway in the Medial Prefrontal Cortex of Rats with Post-traumatic Stress Disorder. Journal of Molecular Neuroscience. 2015;56(1):24-34.

88. Yu B, Wen L, Xiao B, Han F, Shi Y. Single Prolonged Stress induces ATF6 alpha-dependent Endoplasmic reticulum stress and the apoptotic process in medial Frontal Cortex neurons. BMC Neuroscience. 2014;15(1):1.

89. Eagle AL, Fitzpatrick CJ, Perrine SA. Single prolonged stress impairs social and object novelty recognition in rats. Behavioural brain research. 2013;256:591-7.

90. Piao C, Deng X, Wang X, Yuan Y, Liu Z, Liang J. Altered function in medial prefrontal cortex and nucleus accumbens links to stress-induced behavioral inflexibility. Behav Brain Res. 2017;317:16-26.

91. George SA, Rodriguez-Santiago M, Riley J, Abelson JL, Floresco SB, Liberzon I. Alterations in cognitive flexibility in a rat model of post-traumatic stress disorder. Behavioural brain research. 2015;286:256-64.

92. NIMH. Negative Valence Systems: Workshop Proceedings Rockville, Maryland: NIMH; 2011. Available from: https://www.nimh.nih.gov/research-priorities/rdoc/negative-valence-systems-workshop-proceedings.shtml.

93. Forgeard MJ, Haigh EA, Beck AT, Davidson RJ, Henn FA, Maier SF, et al. Beyond Depression: Towards a Process-Based Approach to Research, Diagnosis, and Treatment. Clinical psychology : a publication of the Division of Clinical Psychology of the American Psychological Association. 2011;18(4):275-99.

94. NIMH. Behavioral Assessment Methods for RDoC Constructs. National Advisory Mental Health Council Workgroup on Tasks and Measures for Research Domain Criteria, National Institute of Mental Health, 2016.

95. Sakamoto H, Matsuda K-I, Zuloaga DG, Nishiura N, Takanami K, Jordan CL, et al. Stress affects a gastrin-releasing peptide system in the spinal cord that mediates sexual function: implications for psychogenic erectile dysfunction. PLoS One. 2009;4(1):e4276.

96. Yoshii T, Sakamoto H, Kawasaki M, Ozawa H, Ueta Y, Onaka T, et al. The single-prolonged stress paradigm alters both the morphology and stress response of magnocellular vasopressin neurons. Neuroscience. 2008;156(3):466-74.

97. Liberzon I, Krstov M, Young EA. Stress-restress: effects on ACTH and fast feedback. Psychoneuroendocrinology. 1997;22(6):443-53.

98. Liberzon I, Lopez J, Flagel S, Vazquez D, Young E. Differential regulation of hippocampal glucocorticoid receptors mRNA and fast feedback: relevance to post-traumatic stress disorder. Journal of neuroendocrinology. 1999;11(1):11-7.

99. Cui H, Sakamoto H, Higashi S, Kawata M. Effects of single-prolonged stress on neurons and their afferent inputs in the amygdala. Neuroscience. 2008;152(3):703-12.

100. George SA, Stout SA, Tan M, Knox D, Liberzon I. Early handling attenuates enhancement of glucocorticoid receptors in the prefrontal cortex in an animal model of post-traumatic stress disorder. Biology of mood & anxiety disorders. 2013;3(1):1.

101. Ganon-Elazar E, Akirav I. Cannabinoids and traumatic stress modulation of contextual fear extinction and GR expression in the amygdala-hippocampal-prefrontal circuit. Psychoneuroendocrinology. 2013;38(9):1675-87.

102. Wang H-T, Han F, Shi Y-X. Activity of the 5-HT1A receptor is involved in the alteration of glucocorticoid receptor in hippocampus and corticotropin-releasing factor in hypothalamus in SPS rats. International journal of molecular medicine. 2009;24(2):227.

103. Zhe D, Fang H, Yuxiu S. Expressions of hippocampal mineralocorticoid receptor (MR) and glucocorticoid receptor (GR) in the single-prolonged stress-rats. Acta histochemica et cytochemica. 2008;41(4):89-95.

104. Arai A, Hirota Y, Miyase N, Miyata S, Young LJ, Osako Y, et al. A single prolonged stress paradigm produces enduring impairments in social bonding in monogamous prairie voles. Behavioural Brain Research. 2016;315:83-93.

105. Li M, Han F, Shi Y. Expression of locus coeruleus mineralocorticoid receptor and glucocorticoid receptor in rats under single-prolonged stress. Neurological Sciences. 2011;32(4):625-31.

106. Zhang J-H, Han F, Shi Y-X. Single prolonged stress induces changes in the expression of mineralocorticoid receptor in the medial prefrontal cortex in a rat model of post-traumatic stress disorder. Molecular medicine reports. 2012;6(2):330-4.

107. Sabban EL, Laukova M, Alaluf LG, Olsson E, Serova LI. Locus coeruleus response to single‐prolonged stress and early intervention with intranasal neuropeptide Y. Journal of neurochemistry. 2015;135(5):975-86.

108. Lim S-I, Song K-H, Yoo C-H, Woo D-C, Choe B-Y. Decreased Glutamatergic Activity in the Frontal Cortex of Single Prolonged Stress Model: In vivo and Ex Vivo Proton MR Spectroscopy. Neurochemical Research. 2017:1-12.

109. Knox D, Perrine SA, George SA, Galloway MP, Liberzon I. Single prolonged stress decreases glutamate, glutamine, and creatine concentrations in the rat medial prefrontal cortex. Neuroscience letters. 2010;480(1):16-20.

110. Matsumoto Y, Morinobu S, Yamamoto S, Matsumoto T, Takei S, Fujita Y, et al. Vorinostat ameliorates impaired fear extinction possibly via the hippocampal NMDA-CaMKII pathway in an animal model of posttraumatic stress disorder. Psychopharmacology. 2013;229(1):51-62.

111. Harvey BH, Oosthuizen F, Brand L, Wegener G, Stein DJ. Stress–restress evokes sustained iNOS activity and altered GABA levels and NMDA receptors in rat hippocampus. Psychopharmacology. 2004;175(4):494-502.

112. Harvey BH, Brand L, Jeeva Z, Stein DJ. Cortical/hippocampal monoamines, HPA-axis changes and aversive behavior following stress and restress in an animal model of post-traumatic stress disorder. Physiology & Behavior. 2006;87(5):881-90.

113. George SA, Knox D, Curtis AL, Aldridge JW, Valentino RJ, Liberzon I. Altered locus coeruleus–norepinephrine function following single prolonged stress. European journal of Neuroscience. 2013;37(6):901-9.

114. Harada K, Yamaji T, Matsuoka N. Activation of the serotonin 5-HT 2C receptor is involved in the enhanced anxiety in rats after single-prolonged stress. Pharmacology Biochemistry and Behavior. 2008;89(1):11-6.

115. Luo F-F, Han F, Shi Y-X. Change in 5-HT1A receptor in the dorsal raphe nucleus in a rat model of post-traumatic stress disorder. Mol Med Rep. 2011;4(5):843-7.

116. Liu D, Xiao B, Han F, Luo F, Wang E, Shi Y. Changes in 5-HT1A receptor expression in the oculomotor nucleus in a rat model of post-traumatic stress disorder. Journal of Molecular Neuroscience. 2013;49(2):360-8.

117. Liberzon I, Young EA. Effects of stress and glucocorticoids on CNS oxytocin receptor binding. Psychoneuroendocrinology. 1997;22(6):411-22.

118. Ding J, Han F, Wen L, Xiao B, Shi Y. The role of beta-arrestin-2 on Fear/anxious-related memory in a rat model of Post-traumatic stress disorder. J Affect Disord. 2017;213:1-8.

119. Lee B, Sur B, Yeom M, Shim I, Lee H, Hahm D-H. Effects of systemic administration of ibuprofen on stress response in a rat model of post-traumatic stress disorder. The Korean Journal of Physiology & Pharmacology. 2016;20(4):357-66.

120. Ji LL, Tong L, Peng JB, Jin XH, Wei D, Xu BK, et al. Changes in the expression of the vitamin D receptor and LVSCC‑A1C in the rat hippocampus submitted to single prolonged stress. Molecular medicine reports. 2014;9(4):1165-70.

121. Liu H, Han F, Shi Y. Effect of calreticulin on Ca2+/CaM kinaseIIα and endoplasmic reticulum stress in hippocampal in a rat model of post-traumatic stress disorder. Neurochemical research. 2013;38(7):1407-14.

122. XIAO B, HAN F, SHI Y-x. Expression alteration of CaMKIIα and pCaMKIIα in amygdala neurons in PTSD rats [J]. Progress of Anatomical Sciences. 2009;4:015.

123. Wen Y, Li B, Han F, Wang E, Shi Y. Dysfunction of calcium/calmodulin/CaM kinase IIα cascades in the medial prefrontal cortex in post-traumatic stress disorder. Molecular medicine reports. 2012;6(5):1140-4.

124. Wen L, Han F, Shi Y, Li X. Role of the Endoplasmic Reticulum Pathway in the Medial Prefrontal Cortex in Post-Traumatic Stress Disorder Model Rats. Journal of Molecular Neuroscience. 2016;59(4):471-82.

125. Han F, Yan S, Shi Y. Single-prolonged stress induces endoplasmic reticulum-dependent apoptosis in the hippocampus in a rat model of post-traumatic stress disorder. PloS one. 2013;8(7):e69340.

126. Xie H, Han F, Shi X. Single-prolonged stress induce changes of CaM/CaMKIIα in the rats of dorsal raphe nucleus. Neurochemical research. 2012;37(5):1043-9.

127. Wang H-T, Han F, Gao J-L, Shi Y-X. Increased phosphorylation of extracellular signal-regulated kinase in the medial prefrontal cortex of the single-prolonged stress rats. Cellular and molecular neurobiology. 2010;30(3):437-44.

128. Xiao B, Han F, Wang H-T, Shi Y-X. Single-prolonged stress induces increased phosphorylation of extracellular signal-regulated kinase in a rat model of post-traumatic stress disorder. Mol Med Rep. 2011;4(3):445-9.

129. Liu H, Li H, Xu A, Kan Q, Liu B. Role of phosphorylated ERK in amygdala neuronal apoptosis in single-prolonged stress rats. Molecular medicine reports. 2010;3(6):1059-63.

130. Li XM, Han F, Liu DJ, Shi YX. Single-prolonged stress induced mitochondrial-dependent apoptosis in hippocampus in the rat model of post-traumatic stress disorder. Journal of chemical neuroanatomy. 2010;40(3):248-55.

131. Han F, Xiao B, Wen L. Loss of glial cells of the hippocampus in a rat model of post-traumatic stress disorder. Neurochemical research. 2015;40(5):942-51.

132. Wan J, Liu D, Zhang J, Shi Y, Han F. Single-prolonged stress induce different change in the cell organelle of the hippocampal cells: A study of ultrastructure. Acta histochemica. 2016;118(1):10-9.

133. Ding J, Han F, Shi Y. Single-prolonged stress induces apoptosis in the amygdala in a rat model of post-traumatic stress disorder. Journal of psychiatric research. 2010;44(1):48-55.

134. Xiao B, Yu B, Liu DJ, Han F, Shi YX. Single prolonged stress induces dysfunction of endoplasmic reticulum in a rat model of post-traumatic stress disorder. Molecular medicine reports. 2015;12(2).

135. Li Y, Han F, Shi Y. Changes in integrin αv, vinculin and connexin43 in the medial prefrontal cortex in rats under single-prolonged stress. Molecular medicine reports. 2015;11(4):2520-6.

136. Zhao W, Han F, Shi Y. IRE1α pathway of endoplasmic reticulum stress induces neuronal apoptosis in the locus coeruleus of rats under single prolonged stress. Progress in Neuro-Psychopharmacology and Biological Psychiatry. 2016;69:11-8.

137. Liu D, Xiao B, Han F, Wang E, Shi Y. Single-prolonged stress induces apoptosis in dorsal raphe nucleus in the rat model of posttraumatic stress disorder. BMC psychiatry. 2012;12(1):1.

138. Li Y, Han F, Shi Y. Increased neuronal apoptosis in medial prefrontal cortex is accompanied with changes of Bcl-2 and Bax in a rat model of post-traumatic stress disorder. Journal of Molecular Neuroscience. 2013;51(1):127-37.

139. Xiao B, Yu B, Wang H-t, Han F, Shi Y-x. Single-prolonged stress induces apoptosis by activating cytochrome C/caspase-9 pathway in a rat model of post-traumatic stress disorder. Cellular and molecular neurobiology. 2011;31(1):37-43.

140. Zhang J-H, Li M, Han F, Shi Y-X. Single-prolonged stress induces increased caspase-3 and 9 in mPFC in a rat model of post-traumatic stress disorder. Biomedical Research. 2017;28(3).

141. Xie J, Han F, Shi Y. The unfolded protein response is triggered in rat neurons of the dorsal raphe nucleus after single-prolonged stress. Neurochemical research. 2014;39(4):741-7.
